# *N* ^1^-Methylpseudouridine substitution enhances the performance of synthetic mRNA switches in cells

**DOI:** 10.1093/nar/gkaa070

**Published:** 2020-02-24

**Authors:** Callum J C Parr, Shunsuke Wada, Kenjiro Kotake, Shigetoshi Kameda, Satoshi Matsuura, Souhei Sakashita, Soyoung Park, Hiroshi Sugiyama, Yi Kuang, Hirohide Saito

**Affiliations:** 1 Department of Life Science Frontiers, Center for iPS Cell Research and Application, Kyoto University, 53, Kawaharacho, Sakyo-ku, Kyoto 606-8507, Japan; 2 Department of Chemistry, Graduate School of Science, Kyoto University, Kitashirakawa-Oiwakecho, Sakyo-Ku, Kyoto 606-8502, Japan; 3 Department of Chemical and Biological Engineering, Hong Kong University of Science and Technology, Room 5578, Academic Bldg, Clear Water Bay, Kowloon, Hong Kong

## Abstract

Synthetic messenger RNA (mRNA) tools often use pseudouridine and 5-methyl cytidine as substitutions for uridine and cytidine to avoid the immune response and cytotoxicity induced by introducing mRNA into cells. However, the influence of base modifications on the functionality of the RNA tools is poorly understood. Here we show that synthetic mRNA switches containing *N*^1^-methylpseudouridine (m1Ψ) as a substitution of uridine substantially out-performed all other modified bases studied, exhibiting enhanced microRNA and protein sensitivity, better cell-type separation ability, and comparably low immune stimulation. We found that the observed phenomena stem from the high protein expression from m1Ψ containing mRNA and efficient translational repression in the presence of target microRNAs or proteins. In addition, synthetic gene circuits with m1Ψ significantly improve performance in cells. These findings indicate that synthetic mRNAs with m1Ψ modification have enormous potentials in the research and application of biofunctional RNA tools.

## INTRODUCTION

Synthetic mRNAs are attracting growing attention as tools to control cell behavior because of their tuneable nature and low genomic damage. Synthetic RNAs possess novel functions, including protein expression controllers (1,2), immune regulators (2), biomolecule sensors (3), and cell-fate controllers (4,5). Commonly, pseudouridine-5′-triphosphate (Ψ) and 5-methyl-cytidine-5′-triphosphate (m5C) are used to replace UTP (U) and CTP (C) in the mRNA synthesis, because m5C/Ψ mRNAs trigger low-to-no immune responses to the cells, thereby reducing the cytotoxic side-effect of RNA transfection (6–8). However, the influence of modified bases on the function of synthetic RNA is poorly understood. It is unclear whether m5C/Ψ is the most suitable modification of all RNA tools, especially for biomolecular sensors, in which case the sensory function depends on the recognition between the target molecules and the RNA, and RNA-target binding is largely affected by base modifications.

To address this matter, in this paper we investigated the effect of a series of naturally existing base modifications and their combinations on two model synthetic mRNAs: microRNA (miRNA)- and RNA binding protein (RBP)-sensing mRNA switches (9,10). We found that the substitution of U with*N*^1^-methylpseudouridine-5′-triphosphate (m1Ψ) not only bypasses immune response induced cytotoxicity, but also substantially enhances the effectiveness of both types of switches. The m1Ψ substitution induced higher protein expression of mRNA than other modified bases, allowing clearer detection of signals and better fold-change between ON and OFF states of the switches. In addition, the m1Ψ substitution enhanced the sensitivity and performance of synthetic RNA circuit composed of both miRNA- and RBP-sensing switches. The studies in this work set an example for exploring both the functionality and mechanism of base modifications in synthetic mRNAs, revealing m1Ψ as a prominent base substitution of U. Because the effect of m1Ψ was observed in two types of mRNA switches in all cell types examined, we believe that m1Ψ can broadly be applied to the design of synthetic RNAs that modulate various cell functions.

## MATERIALS AND METHODS

### Cells lines

All cells were cultured at 37°C with 5% CO_2_. HeLa, A2780 (DS Pharma), SH-SY5Y (ATCC), NHDF (Lonza) and 293FT cells (Invitrogen) were cultured in the recommended growth media. Feeder-free hiPSC line 201B7 (Ff-201B7) was a kind gift from Dr Masato Nakagawa, Kyoto University (11), and was maintained on iMatrix-511 (Nippi. Inc) coating in complete StemFit (AK03) medium (Ajinomoto).

### dsDNA template generation for *in vitro* transcription

Templates for *in vitro* transcription were generated by fusion PCR using a T7 promoter sequence-containing 5′-UTR, an open reading frame, and a 3′-UTR. The addition of a 120-nucleotide poly-A tail to the mRNAs was encoded on the PCR template with a reverse primer containing a 120-nucleotide poly-T. Alternatively templates for m6A containing mRNAs contains no poly-A tail sequence. The primers and ORFs used for the template generation of all the switches were adapted from previous studies (9–10,12–16).

### Synthesis and purification of mRNAs

All mRNAs were synthesized using MegaScript T7 Kit (ThermoFisher Scientific). Native mRNAs were synthesized with kit-supplied ATP, CTP, UTP, 1:4 premix of GTP and Anti Reverse Cap Analog (TriLink BioTechnologies). Ψ (pseudouridine-5′-triphosphate), m1Ψ (*N*^1^-methylpseudouridine-5′-triphosphate), m6A (*N*^6^-methyladenosine-5′-triphosphate) and m5C (5-methylcytidine-5′-triphosphate) were purchased from TriLink Biotechnologies and used as full substitutions of UTP, ATP or CTP in the reaction to synthesize modified mRNAs. The reaction mixtures were incubated at 37 °C for 4  h and further incubated at 37 °C for  30  min in the presence of TURBO DNase (ThermoFisher Scientific). RNA products were purified with a FavorPrep Blood/Cultured Cells total RNA extraction column (Favorgen Biotech) according to the manufacturer's protocol and then treatment with Antarctic Phosphatase (New England Biolabs) at 37 °C for  30  min. For m6A-containing mRNAs, the polyA tails were added using polyA polymerase (New England Biolabs). The final RNA products were purified using RNeasy MiniElute Cleanup Kit (Qiagen) and stored as 100 ng/μl solution in water at −30 °C.

### Transfection

Cells were seeded into a 24-well plate at 0.5 × 10^5^ cells/well in 0.5 ml/well of the appropriate medium. After 24 h, mRNAs were transfected using 1 μl of Lipofectamine MessengerMAX (Thermo Fisher Scientific) according to the manufacturer's instructions. The exact amount of mRNA used in each experiment is shown in the figure captions and in Supplementary Table S1. Twenty-four hours after the transfection, the cells were analyzed by flow cytometry. Dead cells and debris were removed by front and lateral light scattering signals, and further distinguished by the staining of 7-AAD. hmAG1 and EGFP were detected by blue laser (488 nm) using a 90% cut FITC filter (530/30 nm), tagBFP was detected by violet laser (405 nm) using a Pacific Blue filter (450/40 nm) and iRFP670 was detected by red laser (633 nm) and violet laser using an APC filter (675/25 nm). The fluorescence from the RNA switches was normalized by the fluorescence intensity from the expression of an internal control mRNA.

### Puromycin elimination of undifferentiated iPSCs from cell cultures

The procedure to remove hiPSCs from the cell culture is adapted from our previous research (15). For pure iPSCs cultures, cells were seeded in 24-well plates at densities of 30,000 or 75,000 cells/well in complete StemFit medium with 10 μM ROCK inhibitor. For partially differentiated iPSCs cultures, iPSCs were first differentiated for 7 days in StemFit without basic FGF according to protocol (11) and then seeded in 24-well plates in complete StemFit medium with 10 μM ROCK inhibitor. After 24 h, 30 ng of each 4xmiR-302a-puroR switch (4x indicates four copies of the miRNA binding sequence) was co-transfected with either 30 pmol of control miRNA inhibitor or miR-302a inhibitor following the manufacturer's protocol for Lipofectamine MessengerMAX. After 6 h, the culture medium was replaced with complete StemFit medium containing 2 μg/ml puromycin. After 24 h, the cells were gently washed twice with warm PBS and cultured for a further 7 days in complete StemFit medium. The medium was changed every other day. The presence of residual iPSCs/colonies was visualized using Leukocyte Alkaline Phosphatase Kit (Sigma), following the manufacturer's protocol. Whole well images were stitched from tiled microscope images (Keyence, BZ-X800).

### RT-qPCR

293FT cells were seeded into a 24-well plate at 0.5 × 10^5^ cells/well in 0.5 ml/well of the appropriate medium. After 24 h, 200 ng of each native or base-modified EGFP mRNAs was introduced using 1 μl of Lipofectamine MessengerMAX. The cells lysate was collected at different time points after the transfection, and total RNA was extracted using a Trizol based method. 100 ng of total RNA was used to produce cDNA (TOYOBO ReverTra Ace). The amount of remaining native or modified mRNA was measured and normalized using the standard SYBR Green RT-qPCR protocol with amplification with EGFP-specific primers and human GAPDH-specific primers (GADPH fwd 5′-GGTCGGAGTCAACGGATTTG-3′, rev 5′-TCAGCCTTGACGGTGCCATG-3′; EGFP fwd 5′-GAAGCGCGATCACATGGT-3′, rev 5′-CCATGCCGAGAGTGATCC-3′).  10  ng of cDNA input was amplified with Power SYBR Green PCR Master Mix (ThermoFisher Scientific) for 40 cycles followed by melt-curve analysis. EGFP mRNA was normalized to GADPH. Raw Ct values were analysed using the Pfaffle ΔΔCt method. Degradation graphs (Supplementary Figure S3B) show the relative amount of mRNA to the amount of native mRNA at 1 h.

### *T*_m_ measurement

To determine the melting temperature (*T*_m_) between two nucleic acid strands, we used a short oligonucleotide sequence pair that are adapted from a standard oligonucleotide pair used to calibrate the *T*_m_ analysis by Shimadzu™. The oligonucleotides were synthesized by *in vitro* transcription using the primer set T7_fwd: TAATACGACTCACTATAG and T7_rev: CGCAAAACGCCTATAGTGAGTCGTATTA. The product oligonucleotides were purified by PAGE and gel extraction. The reverse complementary oligonucleotide was purchased from GeneDesign Inc. (Osaka, Japan). UV melting experiments were performed using a JASCO V-650 UV/VIS spectrophotometer equipped with a high-performance temperature controller and micro auto eight-cell holder (JASCO PAC-743R). First, equal molar concentrations of each oligonucleotide (final 4 μM) and its complementary RNA strand were cooled slowly from 85 °C to room temperature using the ProFlex™ PCR system (ThermoFisher Scientific) in buffer containing 20 mM sodium phosphate and 50 mM NaCl, pH 7.0. The melting profiles, taken at temperatures ranging from 15 to 85 °C, were recorded at 260 nm using a scan rate of 0.5 °C/min. *T*_m_ was calculated as the temperature at which the duplexes were half dissociated, and each *T*_m_ was determined by taking the derivatives of the melting curves.

### WST-1 cell viability assay

In Supplementary Figure S6A, 201B7 cells were fully differentiated after 14 days culture in StemFit medium without basic FGF and then seeded in 24-well plates at a density of 70,000 cells/well in complete StemFit medium with 10 μM ROCK inhibitor. Twenty four hours later, the cells were transfected with either control puroR or 4x302a-puroR switch and control or miR-302a-5p inhibitor. After 6 h, the medium was replaced with complete StemFit medium with 2 μg/ml puromycin. After 24 h, the cells were gently washed twice in PBS, then complete StemFit medium with WST-1 (Roche Diagnostics KK) according to the manufacturer's protocol. The colorimetric reading was obtained on the microplate reader infinite M1000 (Tecan Japan Co., Ltd) after 4 h of WST-1 treatment. The viability of cells transfected with control puroR switch and control miRNA inhibitor was set as 100%.

### Electrophoretic mobility shift assay (EMSA)

Bacterial expression plasmids for recombinant U1A and MS2CP protein with His tag were constructed and expressed in *Escherichia coli* as in our previous works (17). Cells were lysed with either BugBuster® Protein Extraction Reagent for U1A or sonication for MS2CP and the target proteins were purified with AKTA system and stored in 50% glycerol at −80 °C. The 2× U1A aptamer sequence (5′-GACAGCAUUGUACCCAGAGUCUGUCCCCAGACAUUGCACCUGGCGCUGUC-3′) (18) and the 2x MS2 aptamer sequence (5′-GGGAACACGAGCGAGATGGGTGATCCTCACCTCGCTCGTGGCAGATGGGTGATCCTCACCTGCTCCC TATAGTGAGTCGTATTACAATGCCT-3′) were synthesized by IVT from dsDNA templates using the MEGAshortscript™ T7 Transcription Kit (Thermo Fisher Scientific) at 37 °C incubation for 4 h, followed by TURBO DNase treatment to remove the template, and clean up with the Monarch^®^ RNA Cleanup Kit (New England Biolabs). A further purification was carried out with 16% denaturing PAGE (8.3 M urea) and subsequent elution from the gel overnight at 37 °C in 600 μl of elution buffer (0.3 M sodium acetate pH 5.2, 0.1% SDS). The eluted RNAs were filtered with a Ultrafree-MC-HV Centrifugal Filters Durapore-PVDF 0.45 μm (Merck), and purified by phenol−chloroform extraction. The ethanol-precipitated pellet was dissolved in water. The final RNA concentration and purity were measured by Nanodrop (Thermo Fisher Scientific). Native UTP was substituted for either m1Ψ or Ψ to make the modified RNAs. 1 μM of RNA aptamer was mixed with 5× binding buffer (U1A: 100 mM HEPES pH 7.5, 400 mM KCl, 100 mM NaCl, 10 mM DTT; MS2CP: 200 mM HEPES pH 7.5, 50 mM NaCl, 30 mM MgCl_2_, 10 mM DTT, 10 mM spermidine), and nuclease-free water was added to make up the volume. The aptamer structures of the RNAs were reconstructed by denaturing at 80 °C for 3 min followed by slow cooling to room temperature and 10 min incubation at room temperature. Protein solution was added and incubated at 4 °C for 30 min. 10 μl of reaction mixture of each condition was examined on 12% native PAGE gel at 4 °C. The gel was stained with SYBR® Green II Nucleic Acid Gel Stain (Lonza) and imaged on a Typhoon FLA-7000 biomolecular imager (Fujifilm).

### Quantification and statistical analysis

Statistical values including the exact *N* and statistical significance are reported in the figure legends. Statistical analysis (standard deviation or standard error) was performed using Excel. Significant differences using Student's t-test was performed on GraphPad. The fitting of derivative reports of the melting curves (Supplementary Figure S5) was performed using Python. The statistical analysis is based on the means generated from at least three independent experiments. FACS dot plots and histograms were produced in Accuri software or FlowJo. The levels of significance (unpaired two-tailed Student's *t*-test) are denoted as *P < 0.05, **P < 0.01, ***P < 0.001.

## RESULTS

### m1Ψ substitution of U improves mRNA switch performance

**Figure 1.**
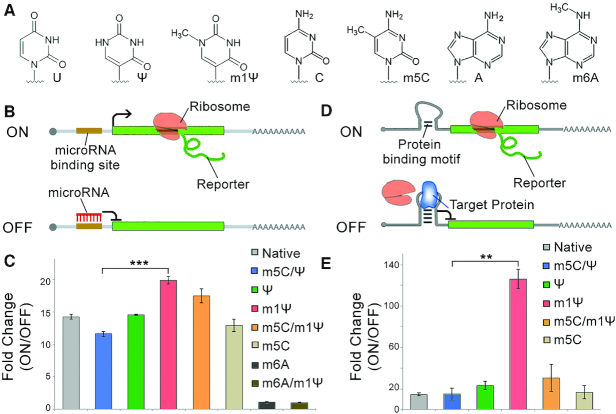
The effect of modified bases in synthetic mRNA switches. (**A**) Chemical structures of the native and modified bases. (**B**) Scheme of the miRNA-sensing switch. miRNA binds to the complementary sequence (miRNA-binding site) residing on the 5′-UTR of the switch to control reporter protein expression. (**C**) Fold-change of miR-21-5p-EGFP switches containing different base modifications in 293FT cells. miR-21-5p mimic was co-transfected to induce OFF state of the switches. Fold-change was analysed based on the level of reporter proteins production from the switches between OFF and ON states. (**D**) Scheme of the RBP-sensing switch. RBP binds to the RNA aptamer (protein binding site) residing on the 5′-UTR of the switch to control reporter protein expression. (**E**) Fold-change of MS2CP- sensing EGFP switches containing different base modifications in 293FT cells. MS2CP-coding mRNA with m5C/Ψ was co-transfected to produce MS2CP and induce OFF state of the switches. Fold-change was analysed based on the level of reporter proteins production from the switches between OFF and ON states. Error bars indicate the mean ± standard error (*n* = 3).

Several types of RNA base modifications are reported to induce low immune response to cells or to impact the protein production rate of the mRNA (Figure 1A) (6–8). These modified nucleotides and their combinations were used in the synthesis (*in vitro* transcription; IVT) of two model mRNA tools: miRNA- and RBP-responsive mRNA switches, of which the translation was regulated upon sensing miRNA or RBP, respectively (9,19). We first used the miR-21-5p- and bacteriophage MS2 coat protein (MS2CP)-responsive switches as representative examples (10,16,20). As shown in Figure 1B, miR-21-5p hinders the translation of miR-21-5p-responsive EGFP switch (bears one miR-21-5p complementary sequence on the 5′UTR of EGFP-coding mRNA), resulting in the suppression of EGFP expression. Because the miR-21-5p expression level in 293FT cells is very low (21), the switch behaves as ON state within 293FT cells. OFF state in 293FT cells is induced by co-transfection with miR-21-5p mimic (22). The difference in EGFP expression between ON and OFF states (denoted as fold-change) represents the sensitivity of the switch for miR-21-5p (Supplementary Figure S1A) (13). Throughout the fluorescence intensity of the switches are normalized by the fluorescence intensity from fluorescent protein produced by co-transfected internal control mRNAs; in this case, iRFP-coding mRNA. As shown in Figure 1C, the m1Ψ-containing switch exhibited the highest fold-change and significantly outperformed the conventional m5C/Ψ switch, suggesting it has the highest sensitivity for miR-21-5p mimic. Similarly, MS2CP protein (16,23) hindered the expression of the MS2CP-sensing EGFP switch (bears MS2CP-binding aptamers on the 5′UTR of the EGFP mRNA), as shown in Figure 1D. Because MS2CP does not exist in mammalian cells, the default of MS2CP-sensing EGFP switch is ON state in 293FT cells. OFF state was induced by co-transfection with MS2CP-coding mRNA. Interestingly, the m1Ψ switch exhibited a substantially higher fold-change than all the other switches, whereas the m5C/Ψ switch exhibited similar fold-change as the native switch (Figure 1E). In addition, we further studied the performance of MS2CP switch with m1Ψ using different amounts of MS2CP-coding mRNA and found that the switch containing m1Ψ consistently showed higher repression efficiency compared with switches containing m5C/Ψ (Supplementary Figure S1B). We focused the following experiments on investigating m1Ψ because of the significant increase of sensitivity to the targets when the mRNA switch was modified with m1Ψ.

### m1Ψ substitution increases basal translation output and decreases immune response

**Figure 2.**
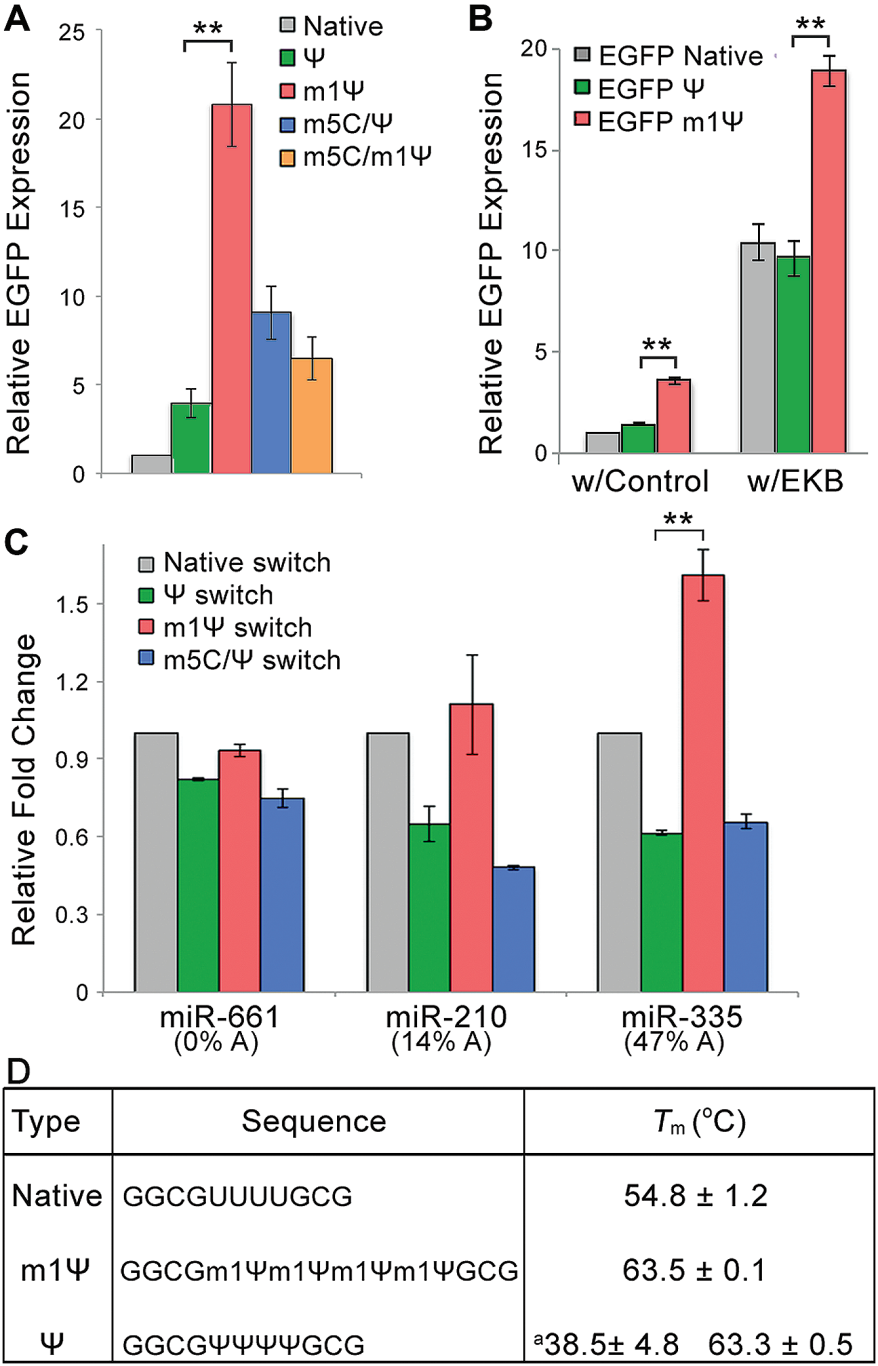
m1Ψ favours mRNA translation and base paring with A. (**A**) Relative EGFP expression in HeLa cells transfected with EGFP mRNAs that carry different base modifications. (**B**) Relative EGFP expression in HeLa cells transfected with EGFP mRNAs with co-transfection of immune-evasive EKB mRNA or control mRNA. (**C**) Relative fold-change of miRNA-sensing switches for miRNA mimics with different A content in 293FT cells. The fold-change of the native switches was set to 1. (**D**) *T*_m_ values of native, Ψ-, or m1Ψ-containing oligos. ^a^*T*_m_ of a possible unstable form was also recorded. Error bars indicate the mean ± standard error (*n* = 3).

We examined the effect of base substitution on the translation of synthetic mRNAs. EGFP expression from m1Ψ-containing miR-21- or MS2CP-sensing switch was higher than that with other modified bases in both 293FT and HeLa cells (Supplementary Figure S2A, B). Similarly, we found that EGFP mRNA with m1Ψ expressed the highest level of EGFP in HeLa cells, confirming that m1Ψ can enhance the translation of mRNAs (Figure 2A). In fact, this phenomenon was universal among all cell types tested, including human induced pluripotent stem cells (hiPSCs 201B7) and cancer cell lines (HeLa, A2780 and SH-SY5Y) (Supplementary Figure S3A). We then examined the cellular degradation rate and transfection efficiency of EGFP mRNAs with different modifications and found no significant differences among the mRNAs, suggesting the enhancement does not stem from these parameters (Supplementary Figure S3B, C). Previous reports suggest the immune response is the key factor for the translation level of Ψ- and m1Ψ-containing mRNAs (7,8). To test whether this theory applies to our observations, we co-transfected EGFP mRNAs with EKB mRNAs (24,25), which has been reported to minimize the immune response due to nucleic acid transfection. The same amount of a control mRNA, which encodes an inert bacterial protein (puromycin resistant protein; puroR), was also co-transfected with EGFP mRNAs to serve as a control. Figure 2B shows that m1Ψ mRNA outperforms native and Ψ-containing mRNAs in EGFP expression even with the EKB co-transfection. Moreover, cells transfected with m1Ψ- or m5C/Ψ-containing mRNAs also showed similar viability (Supplementary Figure S3C). Although the cause of the basal expression increase of m1Ψ-mRNA is not solely due to the reduction of the innate immune response, these results clearly indicate that m1Ψ-mRNA can increase the basal expression and enhance ON expression of mRNA switches.

### m1Ψ substitution increases miRNA sensitivity of mRNA switches

To observe whether m1Ψ has a direct impact on miRNA sensing, we built switches that sense miRNAs with different A content (Figure 2C; the complementary sequences on the switch have different U content). The relative fold-changes of miR-661 (0% A)- and miR-210 (14% A)-responsive switches varied only a small degree with different modified base substitutions. In contrast, m1Ψ miR-335 (47% A)-switch showed significantly higher fold-change than its Ψ counterpart. Although we tested the fold-change of several other A- containing, miRNA-sensing mRNA switches, we failed to find a direct correlation between the number of A in the miRNA and the degree of enhancement (Supplementary Table S2 and Supplementary Figure S4), which we attribute to miRNA binding being highly influenced by the base modifications within the target sequence that binds with the seed sequence and 3' complementary sequence of miRNA over flanking sequences. Nevertheless, we found that m1Ψ-switches are superior to m5C/Ψ-switches in many cases (Supplementary Figure S4), indicating that m1Ψ can directly enhance the switch performance for sensing A-containing miRNAs. We speculate that the enhanced fold-change of m1Ψ-containing switches over the native and Ψ- containing switches comes from several factors, including a higher basal expression of m1Ψ-containing mRNA, lower immuno-response, and enhanced miRNA-sensing. Because the sensing of miRNA involves the recognition and binding between miRNA and the complementary sequence on the switch, we hypothesize that m1Ψ may afford strong binding with A through m1Ψ-A base-paring, allowing the enhanced RNA sensing. We measured the melting temperature (*T*_m_) values of short RNA oligonucleotides (native, Ψ, or m1Ψ) in duplex with a native complementary oligonucleotide. All *T*_m_ values were calculated from the first derivatives of the temperature-dependent UV absorbance (260 nm) profiles (Supplementary Figure S5). As shown in Figure 2D, m1Ψ oligonucleotides gave a substantially higher *T*_m_ than native oligonucleotides, suggesting that m1Ψ oligonucleotides have a higher binding affinity with complementary oligonucleotides, enabling stronger m1Ψ-A base-paring. Interestingly, Ψ oligonucleotides showed two *T*_m_ values, suggesting the existence of an additional unstable binding pattern. This observation, together with the fact that Ψ has an extra *N*^1^ imino proton (26,27) for hydrogen bonding (Supplementary Figure S5), agrees with previous research, which concluded that Ψ can act as a universal base to form wobble base-pairing with multiple bases (28) and may alter structural stability (29). Thus, together with the results above, we conclude that m1Ψ-containing miRNA-sensing switches outperform other switches because m1Ψ allows higher basal protein expression and enhanced miRNA sensitivity.

### m1Ψ substitution increases the performance of cell type identifying synthetic switches

To further increase the miRNA sensitivity, we generated switches that bear 4 copies of miRNA binding sequences (4×), as previous reports suggested that multivalency potentiates the sensitivity of the switch to the target miRNA (12,30). We hypothesized that the number of copies and use of m1Ψ may synergize to boost miRNA sensitivity. A different fluorescent protein (hmAG)-coding ORF was used in order to rule out the possibility of reporter protein bias. We also used HeLa cells instead of 293FT cells to exclude cell-type bias. Because HeLa cells express high levels of miR-21-5p and miR-92a, but low levels of miR-206 and miR-302a (31), inhibitors of miR-21-5p and miR-92a were used to induce ON state of the corresponding switches, while miR-201 and miR-302a mimics were used to induce OFF state for the corresponding switches. The fluorescence of hmAG was normalized by tagBFP fluorescence from co-transfected tagBFP mRNA (internal control). Supplementary Figure S4 shows that for both endogenous and exogenous miRNA-sensing switches, the m1Ψ versions exhibited higher fold-changes than their m5C/Ψ counterparts. These observations indicate that the enhancement of miRNA performance by m1Ψ is independent of cell type and applicable to various A-containing miRNAs.

**Figure 3.**
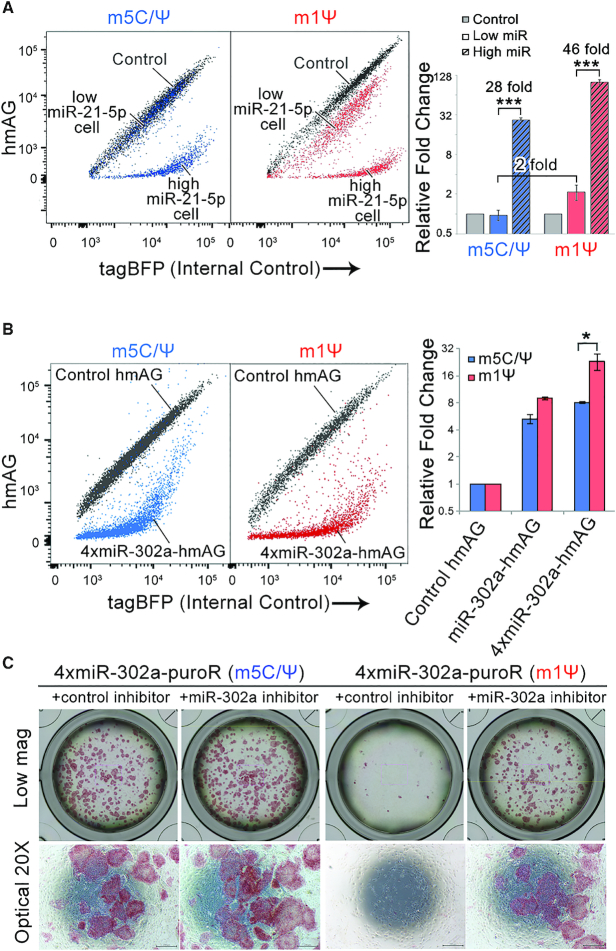
m1Ψ increases the performance of miRNA-sensing switches. (**A**) Representative dot plots showing 293FT and HeLa cells treated with miR-21-5p switch. Fold-differences represent the distance of separation between the two cell populations. (**B**) Representative dot plots and fold-changes of 4xmiR-302a-hmAG switches transfected in hiPSCs (201B7). (**C**) Representative images showing the removal of hiPSCs and partially differentiated hiPSCs via 4xmiR-302a-puroR switch transfection followed by puromycin selection. hiPSCs were visualized by alkaline phosphatase staining. Either control or miR-302a inhibitor was co-transfected with the switch to inhibit the function of endogenous miR-302a in hiPSCs. Error bars indicate the mean ± standard error (*n* = 3).

One of the important applications of miRNA-sensing switches is cell-type recognition and separation. Because each type of cell possesses a unique miRNA profile, miRNA-sensing switches can be used to detect and purify target cells, including differentiated cells derived from hiPSCs (10,15). Thus, we investigated whether m1Ψ-containing switches can improve the resolution of cell separation. We first used a model co-culture containing HeLa cells (high miR-21-5p) and 293FT cells (low miR-21-5p) at a 1-to-1 ratio. By using miR-21 switch with conventional m5C/Ψ, we observed a clear downward shift of the HeLa cell population compared with cells transfected with control hmAG mRNA (Figure 3A, left), but no shift was observed in the 293FT cell population. However, using m1Ψ-containing miR-21 switch, we observed a significantly stronger downward shift in the HeLa cell population and slight shift in the 293FT cell population (Figure 3A, right). This result indicates that m1Ψ-containing switch exhibits higher miRNA sensitivity than m5C/Ψ switch, allowing for both a clearer separation of the two cell populations and the detection of low miRNA activity in 293FT cells.

### m1Ψ substitution increases the effectiveness of autonomous cell elimination switches

Next, we performed selective cell-elimination experiments using miRNA-sensing switches on partially differentiated hiPSCs (201B7, which has high miR-302a expression), because a thorough elimination of undifferentiated and partially differentiated hiPSCs that may form tumors is required before cell transplantation (15). As expected, the m1Ψ-containing 4xmiR-302 switch exhibited stronger suppression of the hmAG expression compared with m5C/Ψ-containing switch in hiPSCs (Figure 3B). We then investigated the effect of m1Ψ on hiPSC elimination using miR-302-Puro switch (15), which encodes a puromycin-resistant protein, puroR. In this experimental setup, upon suppressing the puroR expression with miR-302a, which is highly expressed in hiPSCs, the cell is susceptive to puromycin treatment, which results in cell death. On the other hand, differentiated cells, which have little-to-no miR-302a expression, can express puroR to tolerate puromycin treatment and remain viable. Using the partial differentiation model of our previous study (15), we constructed 4xmiR-302a-puroR switches to remove undifferentiated hiPSCs (11,15). The m1Ψ-containing switch removed residual hiPSCs more robustly than m5C/Ψ-containing switch (Figure 3C), while both switches had little-to-no level of cytotoxicity on the differentiated cells (Supplementary Figure S6A). Notably, we found that although the performance of m5C/Ψ-containing switch for hiPSC elimination was impaired by increased cell density, m1Ψ-containing switch remained effective for eliminating hiPSCs at the highest cell density tested (Supplementary Figure S6B). Taken together, these data confirmed that m1Ψ can enhance the performance of cell-elimination switches.

### m1Ψ substitution improves the performance of synthetic RNA circuits

**Figure 4.**
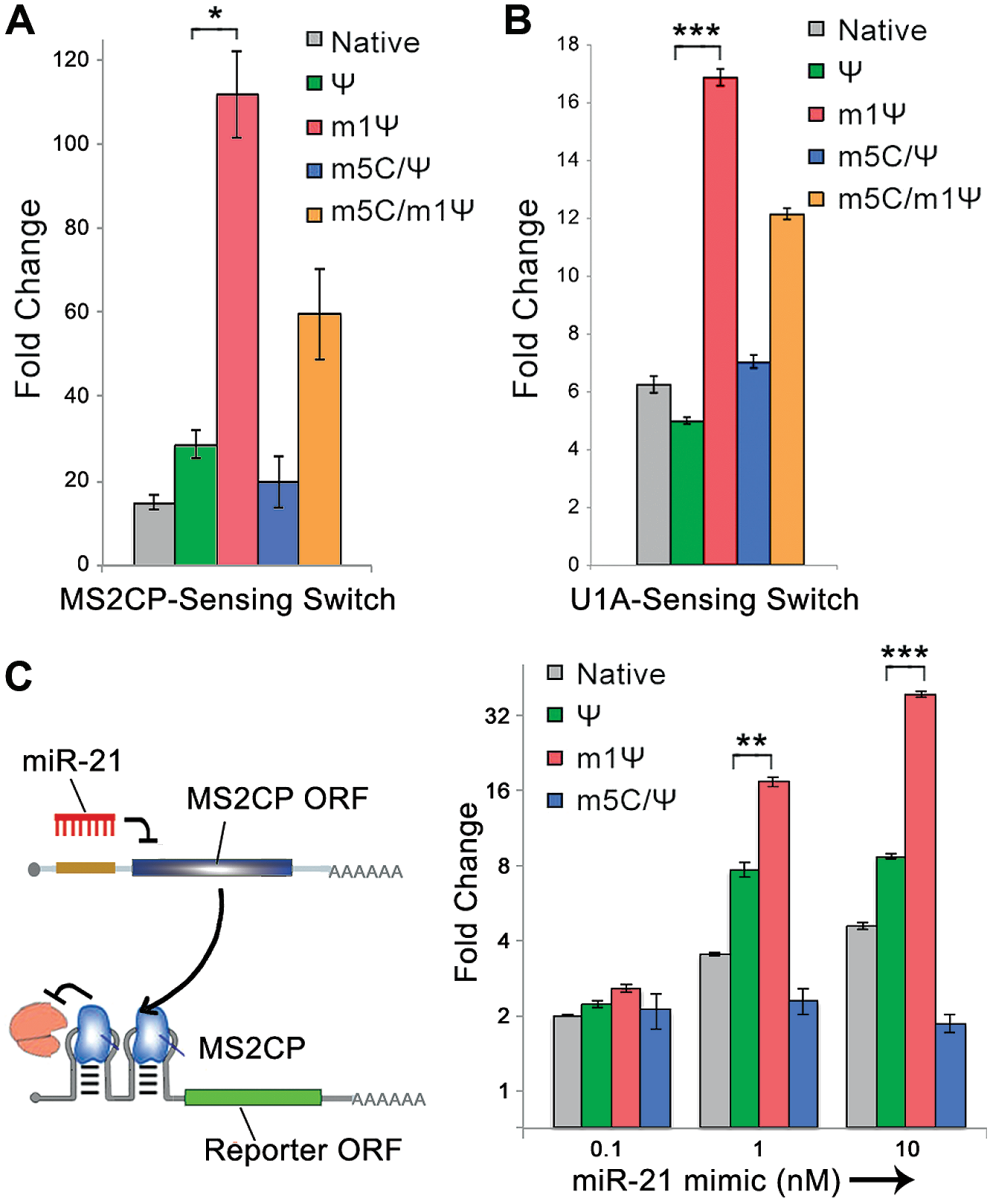
m1Ψ increases the performance of RBP-switches and mRNA circuits. (**A**) Fold-change of MS2CP-responsive EGFP switches carrying different base modifications in HeLa cells. m5C/Ψ-containing MS2CP-coding mRNA was co-transfected to induce OFF state of the switches. (**B**) Fold-change of U1A-responsive EGFP switches carrying different base modifications in 293FT cells. m5C/Ψ-containing U1A-coding mRNA was co-transfected to induce OFF state of the switches. (**C**) Scheme and fold-change of mRNA circuits carrying different base modifications in 293FT cells. miR-21-5p mimic was co-transfected to induce ON state of the circuits. Error bars indicate the mean ± standard error (*n* = 3).

We confirmed that the enhancement of MS2CP-sensing switches with m1Ψ is independent of cell type. MS2CP-sensing switch with m1Ψ showed a higher fold-change over all other switches in HeLa cells (Figure 4A), consistent with our 293FT cell data (Figure 1E). We next examined an alternative RBP, U1A protein, -sensing switch. Similar to MS2CP-switch, m1Ψ containing U1A-switch exhibited a higher fold-change than Ψ-containing U1A-switch (Figure 4B). We observed higher EGFP expression from m1Ψ containing mRNA in ON state and efficiently repressed translation in OFF state (Supplementary Figure S7). We performed an electrophoretic mobility shift assay (EMSA) to examine the effect of base modifications on RBP-aptamer binding *in vitro* (Supplementary Figure S8). Both U1A and MS2CP experiments showed that native U- and m1Ψ-containing 2× aptamers exhibited similar RBP binding affinities, while Ψ-containing 2× aptamer exhibited relatively weaker U1A binding affinity. Because native RNAs induce strong innate immune responses, m1Ψ-containing RNA tools are preferred for the protein-sensing in cells. Moreover, because the binding between an RBP and RNA aptamer is often dependent on the stability of the aptamer structure (32), and both MS2 and U1A aptamers contain U–A pairing on their stems, m1Ψ-A base pairing may stabilize the secondary structures of the aptamers to favour RBP-aptamer binding in the cellular environment. Lastly, we investigated whether m1Ψ can improve the performance of synthetic mRNA circuits composed of both the miRNA- and RBP-sensing switches (Figure 4C, Supplementary Figure S2C) (12). In the presence of miR-21-5p, MS2CP expression was suppressed and EGFP expression was enhanced. On the other hand, in the absence of miR-21-5p, the translation of EGFP mRNA was hindered by MS2CP. As shown in Figure 4C, the m1Ψ- containing circuit gives the highest fold-change in 293FT cells. These observations demonstrated that m1Ψ can greatly enhance the performance of RNA circuit through a synergistic effect from the improved sensitivity towards both miRNA and RBP.

## DISCUSSION

In conclusion, we screened out a base modification, m1Ψ, which can greatly enhance the performance of miRNA- and RBP-responsive mRNA switches. The higher sensitivity of m1Ψ-containing switches for miRNA and RBP (U1A and MS2) allows clear separation of positive and negative miRNA- or RBP-expressing cells and improves the performance of mRNA-based circuits. Therefore, the simple substitution of U for m1Ψ can expand the applicability of artificial mRNA switches to solving real-world issues. For example, m1Ψ modification allows cell-purification switches to thoroughly eliminate residual hiPSCs before transplantation, which may promote the clinical transition of hiPSC-based cell therapies (Figure 3). Previous studies on base modifications in synthetic mRNAs tend to focus on the innate immune response (6–8,33–34), because cell death caused by severe immune response will hamper the *in vivo* application of the synthetic mRNAs. Our observations revealed that m1Ψ enables better fold-change between ON and OFF states of mRNA switches due to the increased basal protein expression at ON state, and enhanced sensitivity towards target miRNAs and RBPs at OFF state. Besides the enhanced basal protein expression, stronger m1Ψ-A base-pairing and the lowered innate immune response, other mechanisms might also contribute to the improved switch performance by m1Ψ. Moreover, although outside the scope of this paper, the correlation between the effect of m1Ψ with the position of U on the miRNA binding sequence (35) and on the aptamer remains an interesting topic to be explored in the future. Based on our findings in this paper and the recent use of a m1Ψ-containing mRNA vaccine *in vivo* (higher protein production level and low immune induction) (36), we propose that m1Ψ should be broadly applicable to other types of synthetic RNA tools. Lastly, as a naturally existing modified base in both prokaryotic (37) and eukaryotic cells (38), our findings imply that m1Ψ might play unique roles in cells.

## Supplementary Material

gkaa070_Supplemental_File

## Notes

Present address: Callum J.C. Parr, Laboratory for Advanced Genomics Circuit, Center for Integrative Medical Sciences, RIKEN, 1-7-22 Suehiro-cho, Tsurumi-ku, Yokohama, Kanagawa 230-0045, Japan; Email: callum.parr@riken.jp.
