# Supplementary material for: N 1-Methylpseudouridine substitution enhances the performance of synthetic mRNA switches in cells
Source: Nucleic Acids Res. 2020 Feb 24;48(6):e35. doi: 10.1093/nar/gkaa070 (PMC7102939; doi:10.1093/nar/gkaa070)
Supplement: gkaa070_Supplemental_File [file gkaa070_supplemental_file.docx]

***N*^1^-Methylpseudouridine Substitution Enhances the Performance of Synthetic mRNA Switches in Cells**

Callum J.C. Parr^1,†^, Shunsuke Wada^1,†^, Kenjiro Kotake^1^, Shigetoshi Kameda^1^, Satoshi Matsuura^1^, Souhei Sakashita^2^, Soyoung Park^2^, Hiroshi Sugiyama^2^, Yi Kuang^3,*^ and Hirohide Saito^1,*^

Supplementary table S1

Supplementary table S2

Supplementary figures (S1-S8)

Supplementary sequences

Supplementary references

**Supplementary Table**

**Supplementary table S1.** Transfection overview

| **Figure** | **mRNA switch** | **Control mRNA** | **Analyte/inhibitor** | **Machine** |
| --- | --- | --- | --- | --- |
| Figures 1C & S2A, B | 90 ng  (EGFP) | m5C/Ψ; 90 ng  (iRFP) | miR mimic; 1 pmol | BD Accuri  C6 |
| Figure S1A | 0, 45, 90, 180, 360 ng  (EGFP) | m5C/Ψ(iRFP); 90 ng  m5C/Ψ; (Click Beetle Luciferase (G68)),  360, 315, 270, 180, 0 ng | miR mimic; 1 pmol | BD Accuri C6 |
| Figure S3A, B | 200 ng  (EGFP) | N/A | N/A | BD Accuri C6 |
| Figures 1E, 4A, S7 | 200 ng  (EGFP) | m5C/Ψ; 90 ng  (iRFP) | m5C/Ψ MS2CP mRNA; 100 ng | BD Accuri C6 |
| Figures 2A & S1B | 150 ng  (EGFP) | m5C/Ψ; 90 ng  (iRFP) | N/A | BD Accuri C6 |
| Figure 2B | 150 ng  (EGFP) | EKB mRNA cocktail or m5C/Ψ puroR mRNA; 100 ng | N/A | BD Accuri C6 |
| Figure 2C | 90 ng  (EGFP) | m5C/Ψ; 90 ng  (iRFP) | miR mimic; 1 pmol | BD Accuri C6 |
| Figure 3A, B | 90 ng  (hmAG) | Matching modification; 180 ng (tagBFP) | Endogenous miRNA | BD Aria II |
| Figures 3C & S6 | 30 ng  (puroR) | N/A | miR inhibitor; 30 pmol | Keyence All-in-One Fluorescence Microscope BZ-X800 |
| Figure 4B & S7 | 90 ng  (EGFP) | m5C/Ψ; 90 ng | m5C/Ψ U1A mRNA; 100 ng | BD Accuri C6 |
| Figure 4C & S2C | 100 ng  (EGFP) | m5C/Ψ;100 ng | miR mimic; 0.1, 1, or 10 pmol | BD Accuri C6 |
| Figure S4 | 90 ng  (hmAG) | Matching modification; 180 ng (tagBFP) | miR mimic; 1 pmol | BD Aria II |

**Supplementary Table S2**.

MicroRNA sequences and their complementary sequences.

U in the complementary sequence is indicated in bold. Seed sequences and their complementary sequences are shown by underscore; 3’ compensatory sites and their complementary sequences are shown by dotted underscore.

| **microRNA** | **microRNA ID (miRBase v21)** | **Sequence** | **Complementary Sequence** |
| --- | --- | --- | --- |
| miR-661 | MIMAT0003324 | ugccugggucucuggccugcgcgu | acgcgcaggccagagacccaggca |
| miR-210-3p | MIMAT0000267 | cugugcgugugacagcggcuga | **u**cagccgc**u**g**u**cacacgcacag |
| miR-335-5p | MIMAT0000765 | ucaagagcaauaacgaaaaaugu | aca**uuuuu**cg**uu**a**uu**gc**u**c**uu**ga |
| miR-21-5p | MIMAT0000076 | uagcuuaucagacugauguuga | **u**caaca**u**cag**u**c**u**ga**u**aagc**u**a |
| miR-302a-5p | MIMAT0000683 | a**cuuaaac**guggauguacuugcu | agcaaguacauccac**guuuaag**u |
| miR-92a-1-5p | MIMAT0004507 | agguugggaucgguugcaaugcu | agca**uu**gcaaccga**u**cccaacc**u** |
| miR-206 | MIMAT0000462 | uggaauguaaggaagugugugg | ccacacac**uu**cc**uu**aca**uu**cca |
| miR-17-5p | MIMAT0000070 | caaagugcuuacagugcagguag | ct**u**cctgc**u**ctgt**uu**gc**u**ctttg |

**Supplementary Figures**

**
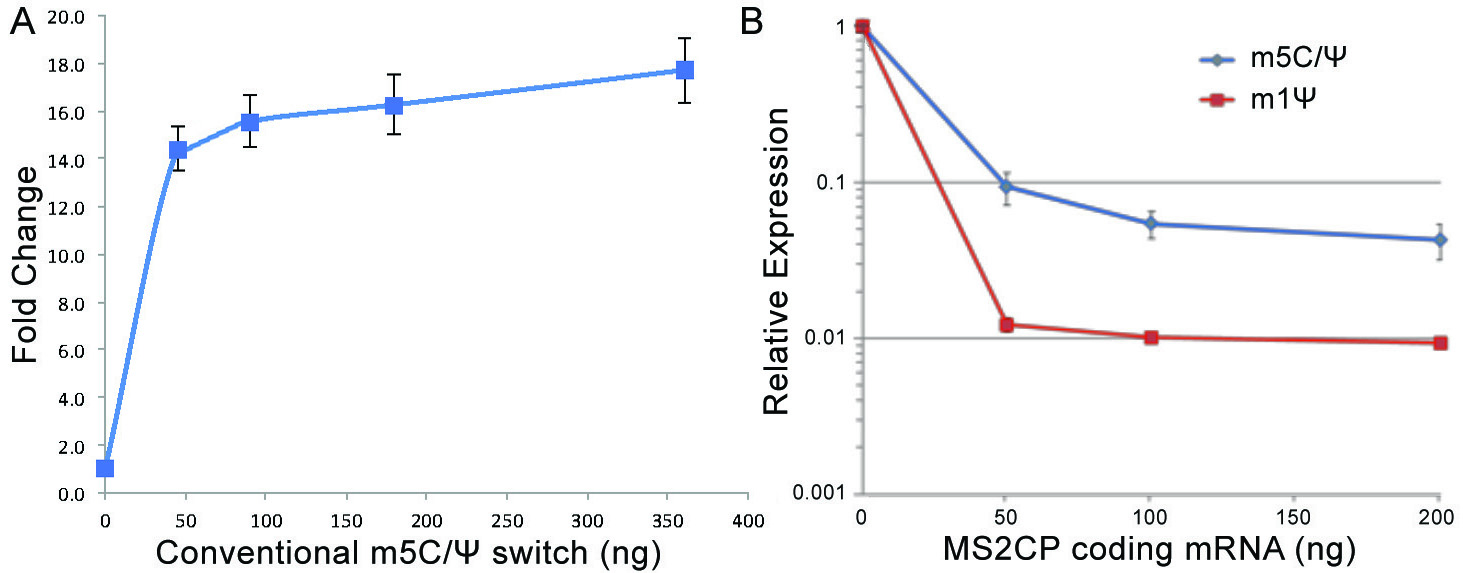
**

**Figure S1. Characterization of mRNA switches.** (A) Dose-response fold-change curve of conventional m5C/Ψ miR-21-5p-responsive switch in 293FT cells co-transfected with 1 pmol of miR-21-5p mimic or negative control mimic. Click Beetle Luciferase mRNA is co-transfected at reverse gradient concentrations to maintain a same total amount of transfected mRNAs among all samples. (B) Dose-response curve of MS2CP-responsive switch with MS2CP mRNA in 293FT cells. Relative switch expression is normalized to the condition without MS2CP mRNA. Results show the mean ± SD (n=3).


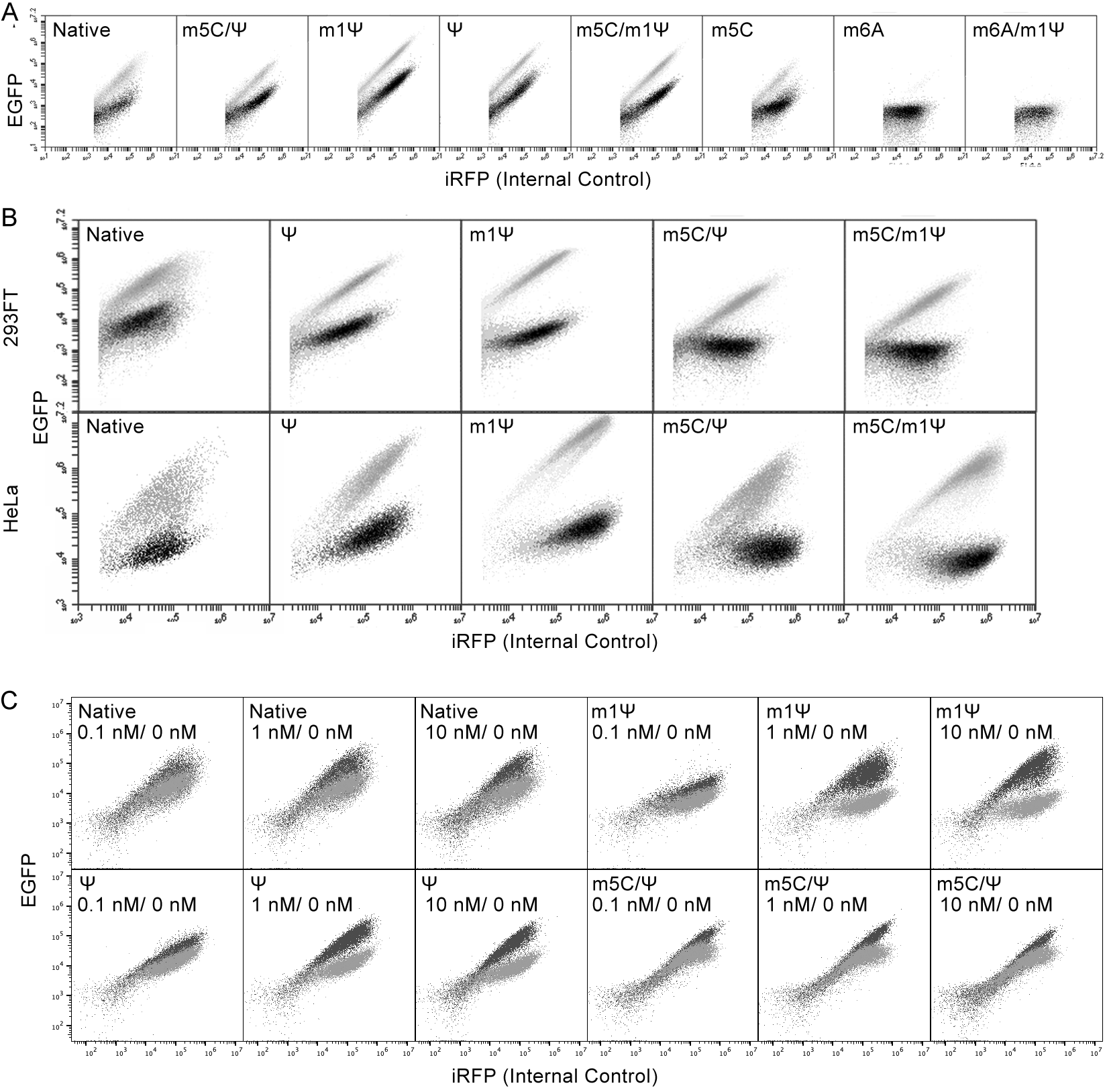


**Figure S2**. (A) Representative dot plots showing the expression of EGFP from miR-21-5p-responsive EGFP mRNAs with different base modifications and the expression of iRFP from internal control iRFP mRNAs in 293FT cells. Gray dots show the expression from cells without co-transfection of miR-21-5p mimic; black dots show the expression from cells with co-transfection of miR-21-5p mimic. (B) Representative dot plots showing the expression of EGFP from MS2CP-responsive EGFP mRNAs with different base modifications and the expression of iRFP from internal control iRFP mRNAs in 293FT and HeLa cells. Gray dots show the expression from cells without co-transfection of MS2CP-coding mRNA; black dots show the expression from cells with co-transfection of MS2CP-coding mRNA. (C) Representative dot plots showing the expression of EGFP from miR-21-5p-responsive mRNA circuits (Fig. 4C) with different base modifications and the expression of iRFP from internal control iRFP mRNAs in 293FT cells. Gray dots show the expression from cells without co-transfection of miR-21-5p mimic; black dots show the expression from cells with co-transfection of miR-21-5p mimic.


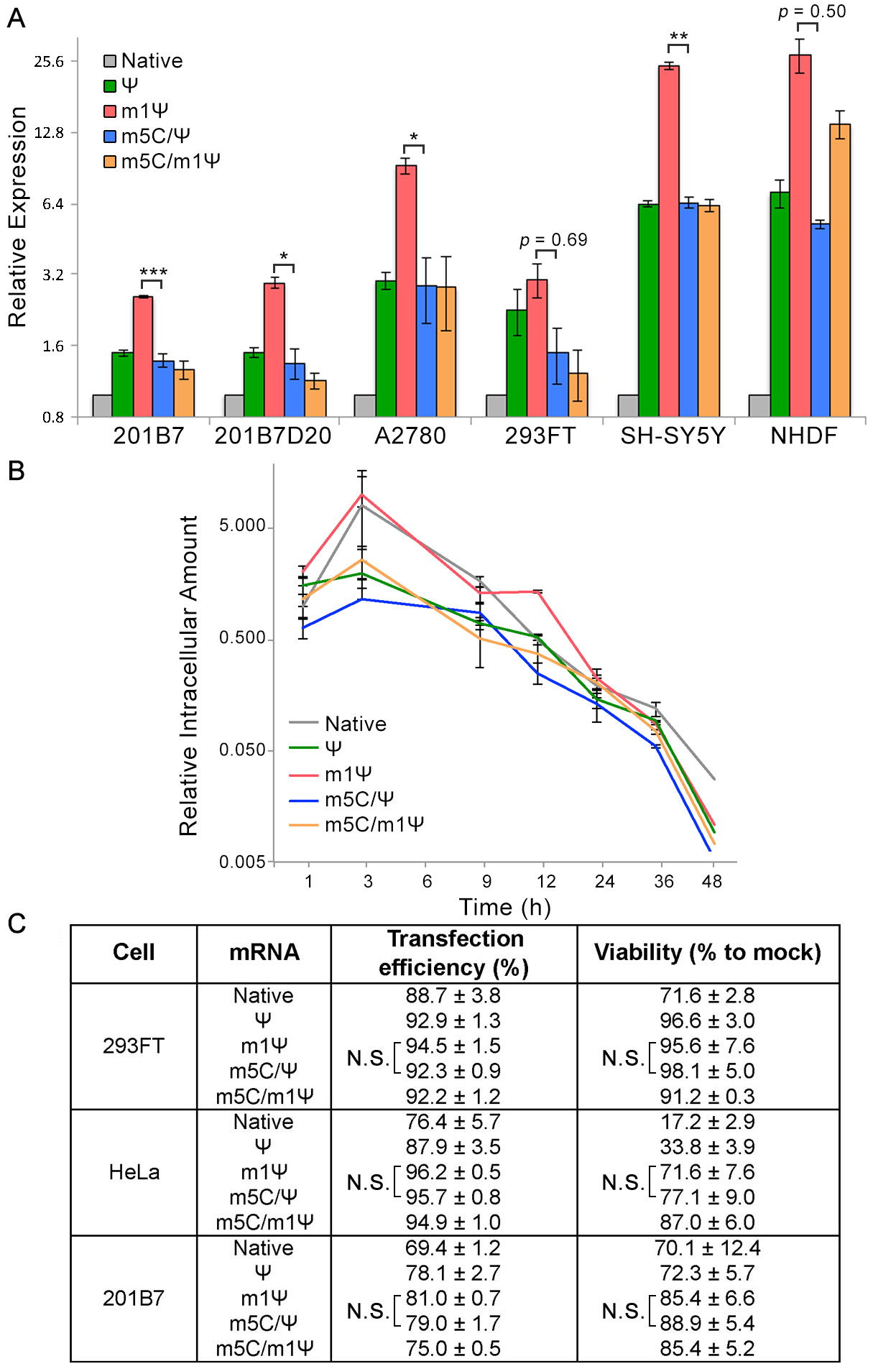


**Figure S3**. (A) Relative EGFP expression from various types of cells transfected with EGFP mRNAs that carry different base modifications. 201B7D20 was fully differentiated 201B7 made by culturing in growth factor free medium for 20 days. n = 3; data are presented as the mean ± SE. (B) qPCR measurements of the degradation curve of different EGFP mRNAs in 293FT cells. X-axis represents time after the transfection of EGFP mRNA. n = 3; data are presented as the mean ± SE. (C) Transfection efficiency and cell viability of different cells after the transfection of different EGFP mRNA. n = 3; data are presented as the mean ± SE.

**
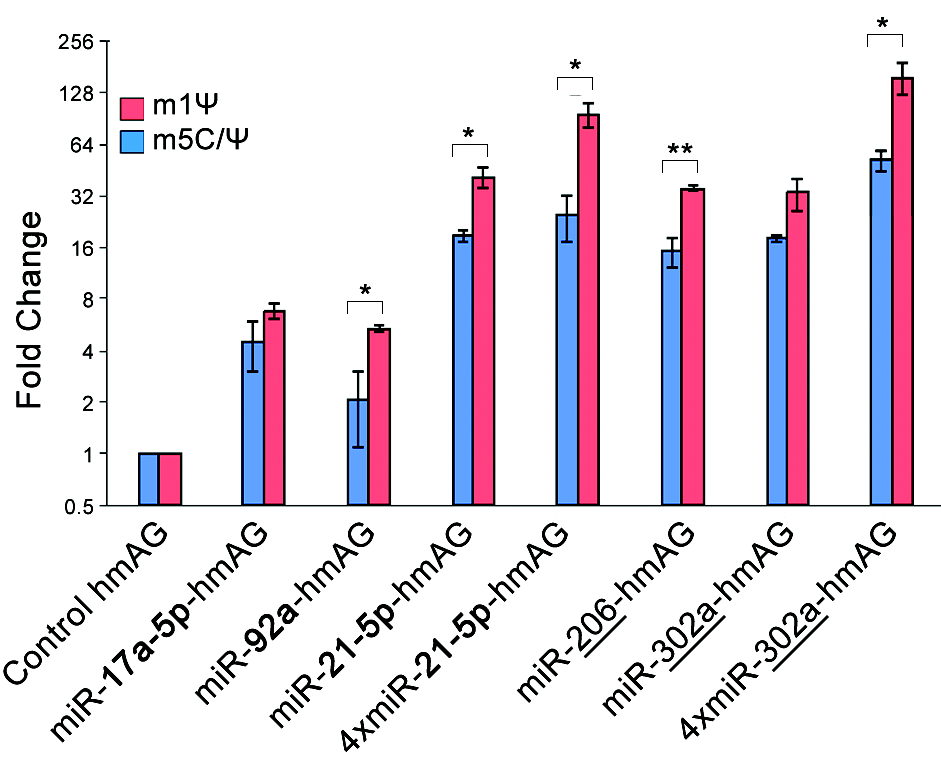
**

**Figure S4**. Fold-change of various kinds of miRNA-hmAG switches in HeLa cells. Fold-changes of endogenous miRNA (shown in bold)-responsive switches were calculated as (T.E. of hmAG with co-transfection of miRNA inhibitor)/(T.E. of hmAG). Fold-changes of non-endogenous miRNA (shown with underscore)-responsive switches were calculated as (T.E. of hmAG)/(T.E. of hmAG with co-transfection of control or corresponding miRNA mimic). n ≥ 3; data are presented as the mean ± SE. T.E., translation efficiency.


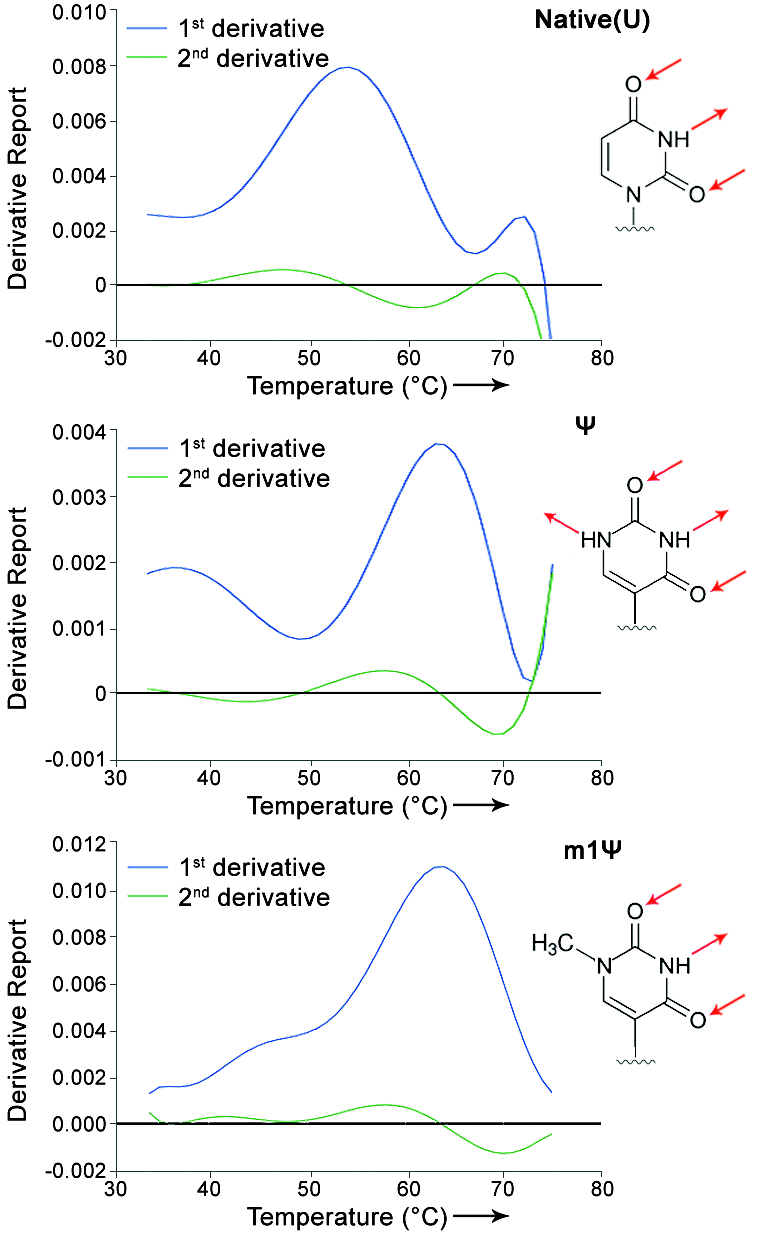


**Figure S5**. Typical first and second derivative reports of the melting curves of native, Ψ-, and m1Ψ-containing oligos to measure *T*_m_. Red arrows show possible hydrogen binding sites on the bases.

**
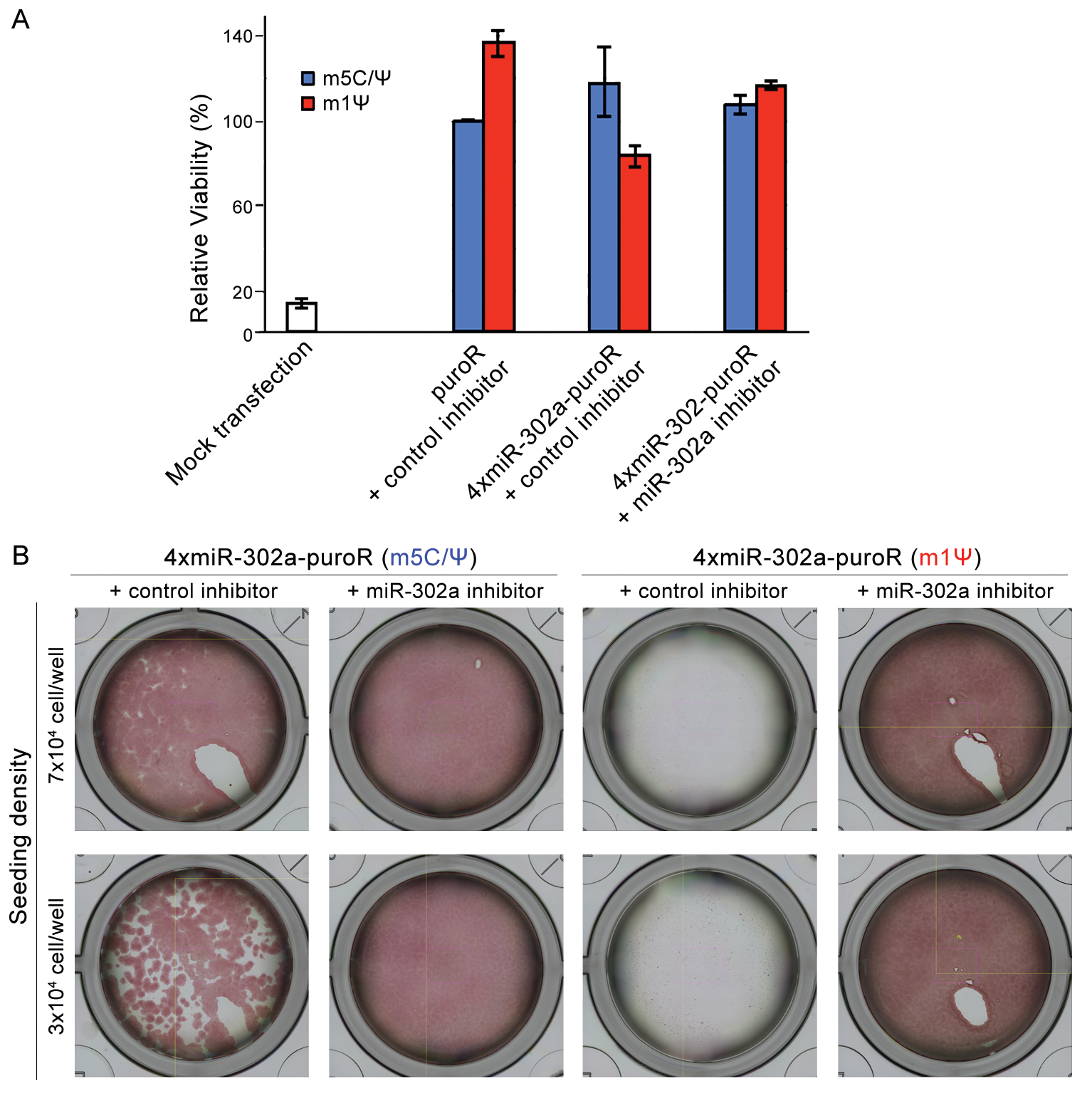
**

**Figure S6**. (A) Relative viability of fully differentiated cells from hiPSCs (201B7, day 14) after 4xmiR-302a-puroR switch transfection followed by puromycin selection. The viability of cells treated with m5C/Ψ puroR mRNA and control inhibitor was set as 100%. n = 3; data are presented as the mean ± SE. (B) Representative images showing the removal of hiPSCs (201B7) via 4xmiR-302a-puroR switch transfection followed by puromycin selection. 201B7 is visualized by alkaline phosphatase staining. The experiments were performed three times, and representative images are shown.


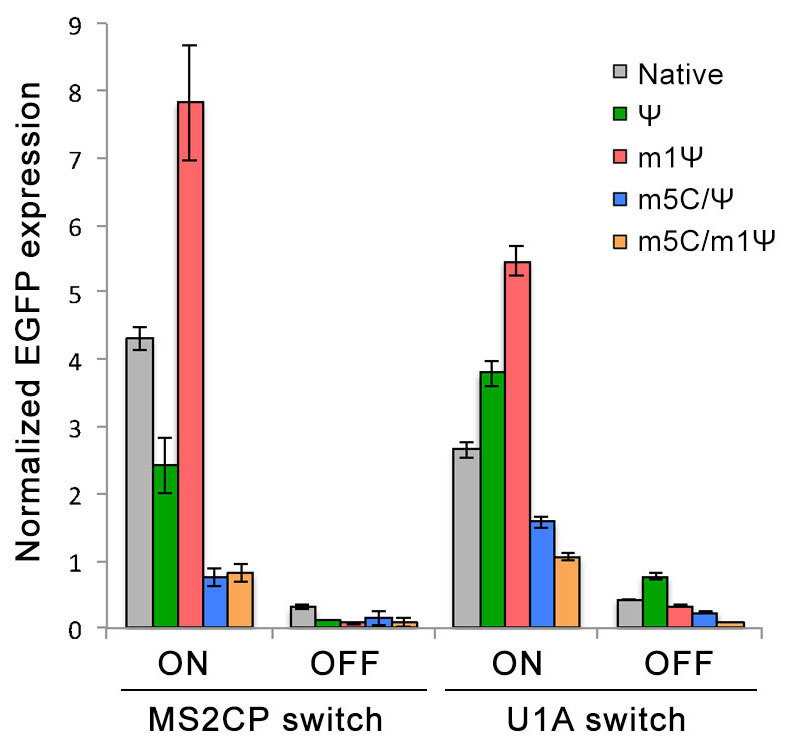


**Figure S7**. Bar graphs showing the EGFP expressions of MS2CP- and U1A-responsive switches at ON and OFF states in 293FT cells. The EGFP expressions are normalized by the expression of co-transfected iRFP mRNA. n = 3; data are presented as the mean ± SE.


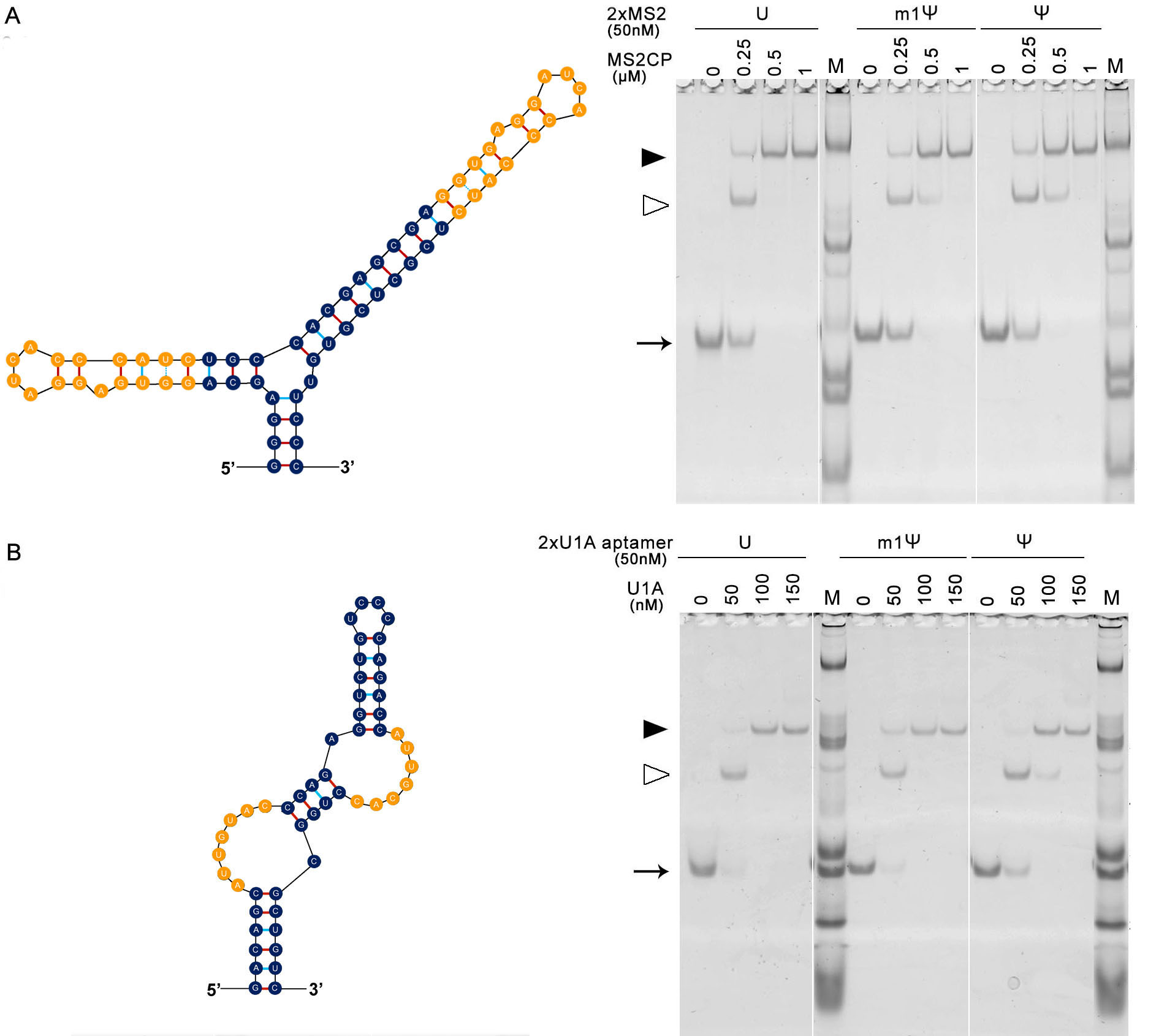


**Figure S8**. Electrophoretic mobility shift assay (EMSA) on RBP with native, Ψ-, and m1Ψ-containing aptamers. (A) EMSA assay of MS2CP with MS2 aptamer. RNA sequence containing 2xMS2 RNA aptamers is adapted from MS2CP-sensing switch. (B) EMSA assay of U1A with U1A-binding aptamer. RNA sequence containing 2xU1A aptamers is adapted from the U1A-sensing switch. The structure of the 2xMS2 aptamer sequence was predicted by Centroid fold (1), and the 2 x U1A aptamer sequence was predicted by previous reports (2, 3) with orange bases indicating the aptamer sequences. Black arrows point to unbound RNAs; white arrowheads point to RNAs bound with one protein; black arrowheads point to RNAs bound with two proteins. M: Dynamarker Small RNA Ⅱ (BioDynamics Laboratory Inc.).

**Supplementary sequences**

**miRNA-sensing switches**

Control miR-EGFP

GGGCGAAUUAAGAGAGAAAAGAAGAGUAAGAAGAAAUAUAAGACACCGGUCGCCACCAUGGUGAGCAAGGGCGAGGAGCUGUUCACCGGGGUGGUGCCCAUCCUGGUCGAGCUGGACGGCGACGUAAACGGCCACAAGUUCAGCGUGUCCGGCGAGGGCGAGGGCGAUGCCACCUACGGCAAGCUGACCCUGAAGUUCAUCUGCACCACCGGCAAGCUGCCCGUGCCCUGGCCCACCCUCGUGACCACCCUGACCUACGGCGUGCAGUGCUUCAGCCGCUACCCCGACCACAUGAAGCAGCACGACUUCUUCAAGUCCGCCAUGCCCGAAGGCUACGUCCAGGAGCGCACCAUCUUCUUCAAGGACGACGGCAACUACAAGACCCGCGCCGAGGUGAAGUUCGAGGGCGACACCCUGGUGAACCGCAUCGAGCUGAAGGGCAUCGACUUCAAGGAGGACGGCAACAUCCUGGGGCACAAGCUGGAGUACAACUACAACAGCCACAACGUCUAUAUCAUGGCCGACAAGCAGAAGAACGGCAUCAAGGUGAACUUCAAGAUCCGCCACAACAUCGAGGACGGCAGCGUGCAGCUCGCCGACCACUACCAGCAGAACACCCCCAUCGGCGACGGCCCCGUGCUGCUGCCCGACAACCACUACCUGAGCACCCAGUCCGCCCUGAGCAAAGACCCCAACGAGAAGCGCGAUCACAUGGUCCUGCUGGAGUUCGUGACCGCCGCCGGGAUCACUCUCGGCAUGGACGAGCUGUACAAGUAGUCUAGACCUUCUGCGGGGCUUGCCUUCUGGCCAUGCCCUUCUUCUCUCCCUUGCACCUGUACCUCUUGGUCUUUGAAUAAAGCCUGAGUAGGAAAAAAAAAAAAAAAAAAAAAAAAAAAAAAAAAAAAAAAAAAAAAAAAAAAAAAAAAAAAAAAAAAAAAAAAAAAAAAAAAAAAAAAAAAAAAAAAAAAAAAAAAAAAAAAAAAAAAAAA

Control-puroR

GGGCGAAUUAAGAGAGAAAAGAAGAGUAAGAAGAAAUAUAAGACACCGGUCGCCACCAUGACCGAGUACAAGCCCACGGUGCGCCUCGCCACCCGCGACGACGUCCCCAGGGCCGUACGCACCCUCGCCGCCGCGUUCGCCGACUACCCCGCCACGCGCCACACCGUCGAUCCGGACCGCCACAUCGAGCGGGUCACCGAGCUGCAAGAACUCUUCCUCACGCGCGUCGGGCUCGACAUCGGCAAGGUGUGGGUCGCGGACGACGGCGCCGCGGUGGCGGUCUGGACCACGCCGGAGAGCGUCGAAGCGGGGGCGGUGUUCGCCGAGAUCGGCCCGCGCAUGGCCGAGUUGAGCGGUUCCCGGCUGGCCGCGCAGCAACAGAUGGAAGGCCUCCUGGCGCCGCACCGGCCCAAGGAGCCCGCGUGGUUCCUGGCCACCGUCGGCGUCUCGCCCGACCACCAGGGCAAGGGUCUGGGCAGCGCCGUCGUGCUCCCCGGAGUGGAGGCGGCCGAGCGCGCCGGGGUGCCCGCCUUCCUGGAGACCUCCGCGCCCCGCAACCUCCCCUUCUACGAGCGGCUCGGCUUCACCGUCACCGCCGACGUCGAGGUGCCCGAAGGACCGCGCACCUGGUGCAUGACCCGCAAGCCCGGUGCCUGAUCUAGACCUUCUGCGGGGCUUGCCUUCUGGCCAUGCCCUUCUUCUCUCCCUUGCACCUGUACCUCUUGGUCUUUGAAUAAAGCCUGAGUAGGAAAAAAAAAAAAAAAAAAAAAAAAAAAAAAAAAAAAAAAAAAAAAAAAAAAAAAAAAAAAAAAAAAAAAAAAAAAAAAAAAAAAAAAAAAAAAAAAAAAAAAAAAAAAAAAAAAAAAAAA

tagBFP

GGGCGAAUUAAGAGAGAAAAGAAGAGUAAGAAGAAAUAUAAGACACCGGUCGCCACCAUGGGAUCCAGCGAGCUGAUUAAGGAGAACAUGCACAUGAAGCUGUACAUGGAGGGCACCGUGGACAACCAUCACUUCAAGUGCACAUCCGAGGGCGAAGGCAAGCCCUACGAGGGCACCCAGACCAUGAGAAUCAAGGUGGUCGAGGGCGGCCCUCUCCCCUUCGCCUUCGACAUCCUGGCUACUAGCUUCCUCUACGGCAGCAAGACCUUCAUCAACCACACCCAGGGCAUCCCCGACUUCUUCAAGCAGUCCUUCCCUGAGGGCUUCACAUGGGAGAGAGUCACCACAUACGAAGACGGGGGCGUGCUGACCGCUACCCAGGACACCAGCCUCCAGGACGGCUGCCUCAUCUACAACGUCAAGAUCAGAGGGGUGAACUUCACAUCCAACGGCCCUGUGAUGCAGAAGAAAACACUCGGCUGGGAGGCCUUCACCGAGACGCUGUACCCCGCUGACGGCGGCCUGGAAGGCAGAAACGACAUGGCCCUGAAGCUCGUGGGCGGGAGCCAUCUGAUCGCAAACAUCAAGACCACAUAUAGAUCCAAGAAACCCGCUAAGAACCUCAAGAUGCCUGGCGUCUACUAUGUGGACUACAGACUGGAAAGAAUCAAGGAGGCCAACAACGAGACCUACGUCGAGCAGCACGAGGUGGCAGUGGCCAGAUACUGCGACCUCCCUAGCAAACUGGGGCACAGAUCUCAUAUGCAUCUCGAGUGAUAGUCUAGACCUUCUGCGGGGCUUGCCUUCUGGCCAUGCCCUUCUUCUCUCCCUUGCACCUGUACCUCUUGGUCUUUGAAUAAAGCCUGAGUAGGAAAAAAAAAAAAAAAAAAAAAAAAAAAAAAAAAAAAAAAAAAAAAAAAAAAAAAAAAAAAAAAAAAAAAAAAAAAAAAAAAAAAAAAAAAAAAAAAAAAAAAAAAAAAAAAAAAAAAAAA

Control Click Beetle Luciferase (G68)

GGGCGAAUUAAGAGAGAAAAGAAGAGUAAGAAGAAAUAUAAGACACCGGUCGCCACCAUGGUGAAACGCGAAAAGAACGUGAUCUACGGCCCAGAACCACUGCAUCCACUGGAAGACCUCACCGCUGGUGAGAUGCUCUUCCGAGCACUGCGUAAACAUAGUCACCUCCCUCAAGCACUCGUGGACGUCGUGGGAGACGAGAGCCUCUCCUACAAAGAAUUUUUCGAAGCUACUGUGCUGUUGGCCCAAAGCCUCCAUAAUUGUGGGUACAAAAUGAACGAUGUGGUGAGCAUUUGUGCUGAGAAUAACACUCGCUUCUUUAUUCCUGUAAUCGCUGCUUGGUACAUCGGCAUGAUUGUCGCCCCUGUGAAUGAAUCUUACAUCCCAGAUGAGCUGUGUAAGGUUAUGGGUAUUAGCAAACCUCAAAUCGUCUUUACUACCAAAAACAUCUUGAAUAAGGUCUUGGAAGUCCAGUCUCGUACUAACUUCAUCAAACGCAUCAUUAUUCUGGAUACCGUCGAAAACAUCCACGGCUGUGAGAGCCUCCCUAACUUCAUCUCUCGUUACAGCGAUGGUAAUAUCGCUAAUUUCAAGCCCUUGCAUUUUGAUCCAGUCGAGCAAGUGGCCGCUAUUUUGUGCUCCUCCGGCACCACUGGUUUGCCUAAAGGUGUCAUGCAGACUCACCAGAAUAUCUGUGUGCGUUUGAUCCACGCUCUCGACCCUCGUGUGGGUACUCAAUUGAUCCCUGGCGUGACUGUGCUGGUGUAUCUGCCUUUCUUUCACGCCUUUGGUUUCUCUAUUACCCUGGGCUAUUUCAUGGUCGGCUUGCGUGUCAUCAUGUUUCGUCGCUUCGACCAAGAAGCCUUCUUGAAGGCUAUUCAAGACUACGAGGUGCGUUCCGUGAUCAACGUCCCUUCAGUCAUUUUGUUCCUGAGCAAAUCUCCUUUGGUUGACAAGUAUGAUCUGAGCAGCUUGCGUGAGCUGUGCUGUGGCGCUGCUCCUUUGGCCAAAGAAGUGGCCGAGGUCGCUGCUAAGCGUCUGAACCUCCCUGGUAUCCGCUGCGGUUUUGGUUUGACUGAGAGCACUUCUGCUAACAUCCAUAGCUUGCGAGACGAGUUUAAGUCUGGUAGCCUGGGUCGCGUGACUCCUCUUAUGGCUGCAAAGAUCGCCGACCGUGAGACCGGCAAAGCACUGGGCCCAAAUCAAGUCGGUGAAUUGUGUAUUAAGGGCCCUAUGGUCUCUAAAGGCUACGUGAACAAUGUGGAGGCCACUAAAGAAGCCAUUGAUGAUGAUGGCUGGCUCCAUAGCGGCGACUUCGGUUACUAUGAUGAGGACGAACACUUCUAUGUGGUCGAUCGCUACAAAGAAUUGAUUAAGUACAAAGGCUCUCAAGUCGCACCAGCCGAACUGGAAGAAAUUUUGCUGAAGAACCCUUGUAUCCGCGACGUGGCCGUCGUGGGUAUCCCAGACUUGGAAGCUGGCGAGUUGCCUAGCGCCUUUGUGGUGAAACAACCCGGCAAGGAGAUCACUGCUAAGGAGGUCUACGACUAUUUGGCCGAGCGCGUGUCUCACACCAAAUAUCUGCGUGGCGGCGUCCGCUUCGUCGAUUCUAUUCCACGCAACGUUACCGGUAAGAUCACUCGUAAAGAGUUGCUGAAGCAACUCCUCGAAAAAGCUGGCGGCUGAAUCUAGACCUUCUGCGGGGCUUGCCUUCUGGCCAUGCCCUUCUUCUCUCCCUUGCACCUGUACCUCUUGGUCUUUGAAUAAAGCCUGAGUAGGAAAAAAAAAAAAAAAAAAAAAAAAAAAAAAAAAAAAAAAAAAAAAAAAAAAAAAAAAAAAAAAAAAAAAAAAAAAAAAAAAAAAAAAAAAAAAAAAAAAAAAAAAAAAAAAAAAAAAAAA

miR-661-EGFP (Complementary miRNA binding site in orange)

GGGCGACUCACUAUAGGUUCCGCGAUCGCGGAUCCACGCGCAGGCCAGAGACCCAGGCAAGAUCACCGGUCGCCACCAUGGUGAGCAAGGGCGAGGAGCUGUUCACCGGGGUGGUGCCCAUCCUGGUCGAGCUGGACGGCGACGUAAACGGCCACAAGUUCAGCGUGUCCGGCGAGGGCGAGGGCGAUGCCACCUACGGCAAGCUGACCCUGAAGUUCAUCUGCACCACCGGCAAGCUGCCCGUGCCCUGGCCCACCCUCGUGACCACCCUGACCUACGGCGUGCAGUGCUUCAGCCGCUACCCCGACCACAUGAAGCAGCACGACUUCUUCAAGUCCGCCAUGCCCGAAGGCUACGUCCAGGAGCGCACCAUCUUCUUCAAGGACGACGGCAACUACAAGACCCGCGCCGAGGUGAAGUUCGAGGGCGACACCCUGGUGAACCGCAUCGAGCUGAAGGGCAUCGACUUCAAGGAGGACGGCAACAUCCUGGGGCACAAGCUGGAGUACAACUACAACAGCCACAACGUCUAUAUCAUGGCCGACAAGCAGAAGAACGGCAUCAAGGUGAACUUCAAGAUCCGCCACAACAUCGAGGACGGCAGCGUGCAGCUCGCCGACCACUACCAGCAGAACACCCCCAUCGGCGACGGCCCCGUGCUGCUGCCCGACAACCACUACCUGAGCACCCAGUCCGCCCUGAGCAAAGACCCCAACGAGAAGCGCGAUCACAUGGUCCUGCUGGAGUUCGUGACCGCCGCCGGGAUCACUCUCGGCAUGGACGAGCUGUACAAGUAGUCUAGACCUUCUGCGGGGCUUGCCUUCUGGCCAUGCCCUUCUUCUCUCCCUUGCACCUGUACCUCUUGGUCUUUGAAUAAAGCCUGAGUAGGAAAAAAAAAAAAAAAAAAAAAAAAAAAAAAAAAAAAAAAAAAAAAAAAAAAAAAAAAAAAAAAAAAAAA AAAAAAAAAAAAAAAAAAAAAAAAAAAAAAAAAAAAAAAAAAAAAAAAAAA

miR-210-EGFP

GGGCGACUCACUAUAGGUUCCGCGAUCGCGGAUCCUCAGCCGCUGUCACACGCACAGAGAUCACACCGGUCGCCACCAUGGUGAGCAAGGGCGAGGAGCUGUUCACCGGGGUGGUGCCCAUCCUGGUCGAGCUGGACGGCGACGUAAACGGCCACAAGUUCAGCGUGUCCGGCGAGGGCGAGGGCGAUGCCACCUACGGCAAGCUGACCCUGAAGUUCAUCUGCACCACCGGCAAGCUGCCCGUGCCCUGGCCCACCCUCGUGACCACCCUGACCUACGGCGUGCAGUGCUUCAGCCGCUACCCCGACCACAUGAAGCAGCACGACUUCUUCAAGUCCGCCAUGCCCGAAGGCUACGUCCAGGAGCGCACCAUCUUCUUCAAGGACGACGGCAACUACAAGACCCGCGCCGAGGUGAAGUUCGAGGGCGACACCCUGGUGAACCGCAUCGAGCUGAAGGGCAUCGACUUCAAGGAGGACGGCAACAUCCUGGGGCACAAGCUGGAGUACAACUACAACAGCCACAACGUCUAUAUCAUGGCCGACAAGCAGAAGAACGGCAUCAAGGUGAACUUCAAGAUCCGCCACAACAUCGAGGACGGCAGCGUGCAGCUCGCCGACCACUACCAGCAGAACACCCCCAUCGGCGACGGCCCCGUGCUGCUGCCCGACAACCACUACCUGAGCACCCAGUCCGCCCUGAGCAAAGACCCCAACGAGAAGCGCGAUCACAUGGUCCUGCUGGAGUUCGUGACCGCCGCCGGGAUCACUCUCGGCAUGGACGAGCUGUACAAGUAGUCUAGACCUUCUGCGGGGCUUGCCUUCUGGCCAUGCCCUUCUUCUCUCCCUUGCACCUGUACCUCUUGGUCUUUGAAUAAAGCCUGAGUAGGAAAAAAAAAAAAAAAAAAAAAAAAAAAAAAAAAAAAAAAAAAAAAAAAAAAAAAAAAAAAAAAAAAAAA AAAAAAAAAAAAAAAAAAAAAAAAAAAAAAAAAAAAAAAAAAAAAAAAAAA

miR-335-EGFP

GGGCGACUCACUAUAGGUUCCGCGAUCGCGGAUCCACAUUUUUCGUUAUUGCUCUUGAAGAUCCACCGGUCGCCACCAUGGUGAGCAAGGGCGAGGAGCUGUUCACCGGGGUGGUGCCCAUCCUGGUCGAGCUGGACGGCGACGUAAACGGCCACAAGUUCAGCGUGUCCGGCGAGGGCGAGGGCGAUGCCACCUACGGCAAGCUGACCCUGAAGUUCAUCUGCACCACCGGCAAGCUGCCCGUGCCCUGGCCCACCCUCGUGACCACCCUGACCUACGGCGUGCAGUGCUUCAGCCGCUACCCCGACCACAUGAAGCAGCACGACUUCUUCAAGUCCGCCAUGCCCGAAGGCUACGUCCAGGAGCGCACCAUCUUCUUCAAGGACGACGGCAACUACAAGACCCGCGCCGAGGUGAAGUUCGAGGGCGACACCCUGGUGAACCGCAUCGAGCUGAAGGGCAUCGACUUCAAGGAGGACGGCAACAUCCUGGGGCACAAGCUGGAGUACAACUACAACAGCCACAACGUCUAUAUCAUGGCCGACAAGCAGAAGAACGGCAUCAAGGUGAACUUCAAGAUCCGCCACAACAUCGAGGACGGCAGCGUGCAGCUCGCCGACCACUACCAGCAGAACACCCCCAUCGGCGACGGCCCCGUGCUGCUGCCCGACAACCACUACCUGAGCACCCAGUCCGCCCUGAGCAAAGACCCCAACGAGAAGCGCGAUCACAUGGUCCUGCUGGAGUUCGUGACCGCCGCCGGGAUCACUCUCGGCAUGGACGAGCUGUACAAGUAGUCUAGACCUUCUGCGGGGCUUGCCUUCUGGCCAUGCCCUUCUUCUCUCCCUUGCACCUGUACCUCUUGGUCUUUGAAUAAAGCCUGAGUAGGAAAAAAAAAAAAAAAAAAAAAAAAAAAAAAAAAAAAAAAAAAAAAAAAAAAAAAAAAAAAAAAAAAAAA AAAAAAAAAAAAAAAAAAAAAAAAAAAAAAAAAAAAAAAAAAAAAAAAAAA

miR-21-5p-EGFP (hmAG version, MS2CP version also created by replacing ORF)

GGGCGACUCACUAUAGGUUCCGCGAUCGCGGAUCCUCAACAUCAGUCUGAUAAGCUAAGAUCACACCGGUCGCCACCAUGAUGGUGAGCAAGGGCGAGGAGCUGUUCACCGGGGUGGUGCCCAUCCUGGUCGAGCUGGACGGCGACGUAAACGGCCACAAGUUCAGCGUGUCCGGCGAGGGCGAGGGCGAUGCCACCUACGGCAAGCUGACCCUGAAGUUCAUCUGCACCACCGGCAAGCUGCCCGUGCCCUGGCCCACCCUCGUGACCACCCUGACCUACGGCGUGCAGUGCUUCAGCCGCUACCCCGACCACAUGAAGCAGCACGACUUCUUCAAGUCCGCCAUGCCCGAAGGCUACGUCCAGGAGCGCACCAUCUUCUUCAAGGACGACGGCAACUACAAGACCCGCGCCGAGGUGAAGUUCGAGGGCGACACCCUGGUGAACCGCAUCGAGCUGAAGGGCAUCGACUUCAAGGAGGACGGCAACAUCCUGGGGCACAAGCUGGAGUACAACUACAACAGCCACAACGUCUAUAUCAUGGCCGACAAGCAGAAGAACGGCAUCAAGGUGAACUUCAAGAUCCGCCACAACAUCGAGGACGGCAGCGUGCAGCUCGCCGACCACUACCAGCAGAACACCCCCAUCGGCGACGGCCCCGUGCUGCUGCCCGACAACCACUACCUGAGCACCCAGUCCGCCCUGAGCAAAGACCCCAACGAGAAGCGCGAUCACAUGGUCCUGCUGGAGUUCGUGACCGCCGCCGGGAUCACUCUCGGCAUGGACGAGCUGUACAAGUAGUCUAGACCUUCUGCGGGGCUUGCCUUCUGGCCAUGCCCUUCUUCUCUCCCUUGCACCUGUACCUCUUGGUCUUUGAAUAAAGCCUGAGUAGGAAAAAAAAAAAAAAAAAAAAAAAAAAAAAAAAAAAAAAAAAAAAAAAAAAAAAAAAAAAAAAAAAAAAA AAAAAAAAAAAAAAAAAAAAAAAAAAAAAAAAAAAAAAAAAAAAAAAAAAA

miR-4x21-5p-EGFP (hmAG version also created)

GGGCGACUCACUAUAGGUUCCGCGAUCGCGGAUCCUCAACAUCAGUCUGAUAAGCUAUCAACAUCAGUCUGAUAAGCUAUCAACAUCAGUCUGAUAAGCUAUCAACAUCAGUCUGAUAAGCUAAGAUCACACCGGUCGCCACCAUGGUGAGCAAGGGCGAGGAGCUGUUCACCGGGGUGGUGCCCAUCCUGGUCGAGCUGGACGGCGACGUAAACGGCCACAAGUUCAGCGUGUCCGGCGAGGGCGAGGGCGAUGCCACCUACGGCAAGCUGACCCUGAAGUUCAUCUGCACCACCGGCAAGCUGCCCGUGCCCUGGCCCACCCUCGUGACCACCCUGACCUACGGCGUGCAGUGCUUCAGCCGCUACCCCGACCACAUGAAGCAGCACGACUUCUUCAAGUCCGCCAUGCCCGAAGGCUACGUCCAGGAGCGCACCAUCUUCUUCAAGGACGACGGCAACUACAAGACCCGCGCCGAGGUGAAGUUCGAGGGCGACACCCUGGUGAACCGCAUCGAGCUGAAGGGCAUCGACUUCAAGGAGGACGGCAACAUCCUGGGGCACAAGCUGGAGUACAACUACAACAGCCACAACGUCUAUAUCAUGGCCGACAAGCAGAAGAACGGCAUCAAGGUGAACUUCAAGAUCCGCCACAACAUCGAGGACGGCAGCGUGCAGCUCGCCGACCACUACCAGCAGAACACCCCCAUCGGCGACGGCCCCGUGCUGCUGCCCGACAACCACUACCUGAGCACCCAGUCCGCCCUGAGCAAAGACCCCAACGAGAAGCGCGAUCACAUGGUCCUGCUGGAGUUCGUGACCGCCGCCGGGAUCACUCUCGGCAUGGACGAGCUGUACAAGUAGUCUAGACCUUCUGCGGGGCUUGCCUUCUGGCCAUGCCCUUCUUCUCUCCCUUGCACCUGUACCUCUUGGUCUUUGAAUAAAGCCUGAGUAGGAAAAAAAAAAAAAAAAAAAAAAAAAAAAAAAAAAAAAAAAAAAAAAAAAAAAAAAAAAAAAAAAAAAAA AAAAAAAAAAAAAAAAAAAAAAAAAAAAAAAAAAAAAAAAAAAAAAAAAAA

miR-302a-5p-hmAG (puroR version also created)

GGGCGACUCACUAUAGGUUCCGCGAUCGCGGAUCCAGCAAGUACAUCCACGUUUAAGUAGAUCCACCGGUCGCCACCAUGGUGAGCGUGAUCAAGCCCGAGAUGAAGAUCAAGCUGUGCAUGAGGGGCACCGUGAACGGCCACAACUUCGUGAUCGAGGGCGAGGGCAAGGGCAACCCCUACGAGGGCACCCAGAUCCUGGACCUGAACGUGACCGAGGGCGCCCCCCUGCCCUUCGCCUACGACAUCCUGACCACCGUGUUCCAGUACGGCAACAGGGCCUUCACCAAGUACCCCGCCGACAUCCAGGACUACUUCAAGCAGACCUUCCCCGAGGGCUACCACUGGGAGAGGAGCAUGACCUACGAGGACCAGGGCAUCUGCACCGCCACCAGCAACAUCAGCAUGAGGGGCGACUGCUUCUUCUACGACAUCAGGUUCGACGGCACCAACUUCCCCCCCAACGGCCCCGUGAUGCAGAAGAAGACCCUGAAGUGGGAGCCCAGCACCGAGAAGAUGUACGUGGAGGACGGCGUGCUGAAGGGCGACGUGAACAUGAGGCUGCUGCUGGAGGGCGGCGGCCACUACAGGUGCGACUUCAAGACCACCUACAAGGCCAAGAAGGAGGUGAGGCUGCCCGACGCCCACAAGAUCGACCACAGGAUCGAGAUCCUGAAGCACGACAAGGACUACAACAAGGUGAAGCUGUACGAGAACGCCGUGGCCAGGUACUCCAUGCUGCCCAGCCAGGCCAAGUGAAUCUAGACCUUCUGCGGGGCUUGCCUUCUGGCCAUGCCCUUCUUCUCUCCCUUGCACCUGUACCUCUUGGUCUUUGAAUAAAGCCUGAGUAGGAAAAAAAAAAAAAAAAAAAAAAAAAAAAAAAAAAAAAAAAAAAAAAAAAAAAAAAAAAAAAAAAAAAAAAAAAAAAAAAAAAAAAAAAAAAAAAAAAAAAAAAAAAAAAAAAAAAAAAAA

miR-4x-302a-5p-hmAG (puroR version also created)

GGGCGACUCACUAUAGGUUCCGCGAUCGCGGAUCCAGCAAGUACAUCCACGUUUAAGUAGCAAGUACAUCCACGUUUAAGUAGCAAGUACAUCCACGUUUAAGUAGCAAGUACAUCCACGUUUAAGUAGAUCACACCGGUCGCCACCAUGGUGAGCGUGAUCAAGCCCGAGAUGAAGAUCAAGCUGUGCAUGAGGGGCACCGUGAACGGCCACAACUUCGUGAUCGAGGGCGAGGGCAAGGGCAACCCCUACGAGGGCACCCAGAUCCUGGACCUGAACGUGACCGAGGGCGCCCCCCUGCCCUUCGCCUACGACAUCCUGACCACCGUGUUCCAGUACGGCAACAGGGCCUUCACCAAGUACCCCGCCGACAUCCAGGACUACUUCAAGCAGACCUUCCCCGAGGGCUACCACUGGGAGAGGAGCAUGACCUACGAGGACCAGGGCAUCUGCACCGCCACCAGCAACAUCAGCAUGAGGGGCGACUGCUUCUUCUACGACAUCAGGUUCGACGGCACCAACUUCCCCCCCAACGGCCCCGUGAUGCAGAAGAAGACCCUGAAGUGGGAGCCCAGCACCGAGAAGAUGUACGUGGAGGACGGCGUGCUGAAGGGCGACGUGAACAUGAGGCUGCUGCUGGAGGGCGGCGGCCACUACAGGUGCGACUUCAAGACCACCUACAAGGCCAAGAAGGAGGUGAGGCUGCCCGACGCCCACAAGAUCGACCACAGGAUCGAGAUCCUGAAGCACGACAAGGACUACAACAAGGUGAAGCUGUACGAGAACGCCGUGGCCAGGUACUCCAUGCUGCCCAGCCAGGCCAAGUGAAUCUAGACCUUCUGCGGGGCUUGCCUUCUGGCCAUGCCCUUCUUCUCUCCCUUGCACCUGUACCUCUUGGUCUUUGAAUAAAGCCUGAGUAGGAAAAAAAAAAAAAAAAAAAAAAAAAAAAAAAAAAAAAAAAAAAAAAAAAAAAAAAAAAAAAAAAAAAAAAAAAAAAAAAAAAAAAAAAAAAAAAAAAAAAAAAAAAAAAAAAAAAAAAAA

miR-92a-5p-hmAG

GGGCGACUCACUAUAGGUUCCGCGAUCGCGGAUCCAGCAUUGCAACCGAUCCCAACCUUAGAUCCACCGGUCGCCACCAUGGUGAGCGUGAUCAAGCCCGAGAUGAAGAUCAAGCUGUGCAUGAGGGGCACCGUGAACGGCCACAACUUCGUGAUCGAGGGCGAGGGCAAGGGCAACCCCUACGAGGGCACCCAGAUCCUGGACCUGAACGUGACCGAGGGCGCCCCCCUGCCCUUCGCCUACGACAUCCUGACCACCGUGUUCCAGUACGGCAACAGGGCCUUCACCAAGUACCCCGCCGACAUCCAGGACUACUUCAAGCAGACCUUCCCCGAGGGCUACCACUGGGAGAGGAGCAUGACCUACGAGGACCAGGGCAUCUGCACCGCCACCAGCAACAUCAGCAUGAGGGGCGACUGCUUCUUCUACGACAUCAGGUUCGACGGCACCAACUUCCCCCCCAACGGCCCCGUGAUGCAGAAGAAGACCCUGAAGUGGGAGCCCAGCACCGAGAAGAUGUACGUGGAGGACGGCGUGCUGAAGGGCGACGUGAACAUGAGGCUGCUGCUGGAGGGCGGCGGCCACUACAGGUGCGACUUCAAGACCACCUACAAGGCCAAGAAGGAGGUGAGGCUGCCCGACGCCCACAAGAUCGACCACAGGAUCGAGAUCCUGAAGCACGACAAGGACUACAACAAGGUGAAGCUGUACGAGAACGCCGUGGCCAGGUACUCCAUGCUGCCCAGCCAGGCCAAGUGAAUCUAGACCUUCUGCGGGGCUUGCCUUCUGGCCAUGCCCUUCUUCUCUCCCUUGCACCUGUACCUCUUGGUCUUUGAAUAAAGCCUGAGUAGGAAAAAAAAAAAAAAAAAAAAAAAAAAAAAAAAAAAAAAAAAAAAAAAAAAAAAAAAAAAAAAAAAAAAAAAAAAAAAAAAAAAAAAAAAAAAAAAAAAAAAAAAAAAAAAAAAAAAAAAA

miR-206-hmAG

GGGCGACUCACUAUAGGUUCCGCGAUCGCGGAUCCCCACACACUUCCUUACAUUCCAAGAUCCACCGGUCGCCACCAUGGUGAGCGUGAUCAAGCCCGAGAUGAAGAUCAAGCUGUGCAUGAGGGGCACCGUGAACGGCCACAACUUCGUGAUCGAGGGCGAGGGCAAGGGCAACCCCUACGAGGGCACCCAGAUCCUGGACCUGAACGUGACCGAGGGCGCCCCCCUGCCCUUCGCCUACGACAUCCUGACCACCGUGUUCCAGUACGGCAACAGGGCCUUCACCAAGUACCCCGCCGACAUCCAGGACUACUUCAAGCAGACCUUCCCCGAGGGCUACCACUGGGAGAGGAGCAUGACCUACGAGGACCAGGGCAUCUGCACCGCCACCAGCAACAUCAGCAUGAGGGGCGACUGCUUCUUCUACGACAUCAGGUUCGACGGCACCAACUUCCCCCCCAACGGCCCCGUGAUGCAGAAGAAGACCCUGAAGUGGGAGCCCAGCACCGAGAAGAUGUACGUGGAGGACGGCGUGCUGAAGGGCGACGUGAACAUGAGGCUGCUGCUGGAGGGCGGCGGCCACUACAGGUGCGACUUCAAGACCACCUACAAGGCCAAGAAGGAGGUGAGGCUGCCCGACGCCCACAAGAUCGACCACAGGAUCGAGAUCCUGAAGCACGACAAGGACUACAACAAGGUGAAGCUGUACGAGAACGCCGUGGCCAGGUACUCCAUGCUGCCCAGCCAGGCCAAGUGAAUCUAGACCUUCUGCGGGGCUUGCCUUCUGGCCAUGCCCUUCUUCUCUCCCUUGCACCUGUACCUCUUGGUCUUUGAAUAAAGCCUGAGUAGGAAAAAAAAAAAAAAAAAAAAAAAAAAAAAAAAAAAAAAAAAAAAAAAAAAAAAAAAAAAAAAAAAAAAAAAAAAAAAAAAAAAAAAAAAAAAAAAAAAAAAAAAAAAAAAAAAAAAAAAA

miR-17-5p-hmAG

GGGCGACUCACUAUAGGUUCCGCGAUCGCGGAUCCctucctgcuctgtuugcuctttgAGAUCCACCGGUCGCCACC/AUGGUGAGCGUGAUCAAGCCCGAGAUGAAGAUCAAGCUGUGCAUGAGGGGCACCGUGAACGGCCACAACUUCGUGAUCGAGGGCGAGGGCAAGGGCAACCCCUACGAGGGCACCCAGAUCCUGGACCUGAACGUGACCGAGGGCGCCCCCCUGCCCUUCGCCUACGACAUCCUGACCACCGUGUUCCAGUACGGCAACAGGGCCUUCACCAAGUACCCCGCCGACAUCCAGGACUACUUCAAGCAGACCUUCCCCGAGGGCUACCACUGGGAGAGGAGCAUGACCUACGAGGACCAGGGCAUCUGCACCGCCACCAGCAACAUCAGCAUGAGGGGCGACUGCUUCUUCUACGACAUCAGGUUCGACGGCACCAACUUCCCCCCCAACGGCCCCGUGAUGCAGAAGAAGACCCUGAAGUGGGAGCCCAGCACCGAGAAGAUGUACGUGGAGGACGGCGUGCUGAAGGGCGACGUGAACAUGAGGCUGCUGCUGGAGGGCGGCGGCCACUACAGGUGCGACUUCAAGACCACCUACAAGGCCAAGAAGGAGGUGAGGCUGCCCGACGCCCACAAGAUCGACCACAGGAUCGAGAUCCUGAAGCACGACAAGGACUACAACAAGGUGAAGCUGUACGAGAACGCCGUGGCCAGGUACUCCAUGCUGCCCAGCCAGGCCAAGUGAAUCUAGACCUUCUGCGGGGCUUGCCUUCUGGCCAUGCCCUUCUUCUCUCCCUUGCACCUGUACCUCUUGGUCUUUGAAUAAAGCCUGAGUAGGAAAAAAAAAAAAAAAAAAAAAAAAAAAAAAAAAAAAAAAAAAAAAAAAAAAAAAAAAAAAAAAAAAAAAAAAAAAAAAAAAAAAAAAAAAAAAAAAAAAAAAAAAAAAAAAAAAAAAAAA

**RBP-sensing switches**

EGFP No aptamer

GGGCGAAUUAAGAGAGAAAAGAAGAGUAAGAAGAAAUAUAAGACACCGGUCGCCACCAUGGUGAGCAAGGGCGAGGAGCUGUUCACCGGGGUGGUGCCCAUCCUGGUCGAGCUGGACGGCGACGUAAACGGCCACAAGUUCAGCGUGUCCGGCGAGGGCGAGGGCGAUGCCACCUACGGCAAGCUGACCCUGAAGUUCAUCUGCACCACCGGCAAGCUGCCCGUGCCCUGGCCCACCCUCGUGACCACCCUGACCUACGGCGUGCAGUGCUUCAGCCGCUACCCCGACCACAUGAAGCAGCACGACUUCUUCAAGUCCGCCAUGCCCGAAGGCUACGUCCAGGAGCGCACCAUCUUCUUCAAGGACGACGGCAACUACAAGACCCGCGCCGAGGUGAAGUUCGAGGGCGACACCCUGGUGAACCGCAUCGAGCUGAAGGGCAUCGACUUCAAGGAGGACGGCAACAUCCUGGGGCACAAGCUGGAGUACAACUACAACAGCCACAACGUCUAUAUCAUGGCCGACAAGCAGAAGAACGGCAUCAAGGUGAACUUCAAGAUCCGCCACAACAUCGAGGACGGCAGCGUGCAGCUCGCCGACCACUACCAGCAGAACACCCCCAUCGGCGACGGCCCCGUGCUGCUGCCCGACAACCACUACCUGAGCACCCAGUCCGCCCUGAGCAAAGACCCCAACGAGAAGCGCGAUCACAUGGUCCUGCUGGAGUUCGUGACCGCCGCCGGGAUCACUCUCGGCAUGGACGAGCUGUACAAGUAGUCUAGACCUUCUGCGGGGCUUGCCUUCUGGCCAUGCCCUUCUUCUCUCCCUUGCACCUGUACCUCUUGGUCUUUGAAUAAAGCCUGAGUAGGAAAAAAAAAAAAAAAAAAAAAAAAAAAAAAAAAAAAAAAAAAAAAAAAAAAAAAAAAAAAAAAAAAAAAAAAAAAAAAAAAAAAAAAAAAAAAAAAAAAAAAAAAAAAAAAAAAAAAAAA

scMS2(WTx2)-EGFP (Protein binding motif)

GGUCAGAUCCGCUAGCGGAUCCGGGAGCAGGUGAGGAUCACCCAUCUGCCACGAGCGAGGUGAGGAUCACCCAUCUCGCUCGUGUUCCCACCGGUCGCCACCAUGGUGAGCAAGGGCGAGGAGCUGUUCACCGGGGUGGUGCCCAUCCUGGUCGAGCUGGACGGCGACGUAAACGGCCACAAGUUCAGCGUGUCCGGCGAGGGCGAGGGCGAUGCCACCUACGGCAAGCUGACCCUGAAGUUCAUCUGCACCACCGGCAAGCUGCCCGUGCCCUGGCCCACCCUCGUGACCACCCUGACCUACGGCGUGCAGUGCUUCAGCCGCUACCCCGACCACAUGAAGCAGCACGACUUCUUCAAGUCCGCCAUGCCCGAAGGCUACGUCCAGGAGCGCACCAUCUUCUUCAAGGACGACGGCAACUACAAGACCCGCGCCGAGGUGAAGUUCGAGGGCGACACCCUGGUGAACCGCAUCGAGCUGAAGGGCAUCGACUUCAAGGAGGACGGCAACAUCCUGGGGCACAAGCUGGAGUACAACUACAACAGCCACAACGUCUAUAUCAUGGCCGACAAGCAGAAGAACGGCAUCAAGGUGAACUUCAAGAUCCGCCACAACAUCGAGGACGGCAGCGUGCAGCUCGCCGACCACUACCAGCAGAACACCCCCAUCGGCGACGGCCCCGUGCUGCUGCCCGACAACCACUACCUGAGCACCCAGUCCGCCCUGAGCAAAGACCCCAACGAGAAGCGCGAUCACAUGGUCCUGCUGGAGUUCGUGACCGCCGCCGGGAUCACUCUCGGCAUGGACGAGCUGUACAAGUAGUCUAGACCUUCUGCGGGGCUUGCCUUCUGGCCAUGCCCUUCUUCUCUCCCUUGCACCUGUACCUCUUGGUCUUUGAAUAAAGCCUGAGUAGGAAAAAAAAAAAAAAAAAAAAAAAAAAAAAAAAAAAAAAAAAAAAAAAAAAAAAAAAAAAAAAAAAAAAAAAAAAAAAAAAAAAAAAAAAAAAAAAAAAAAAAAAAAAAAAAAAAAAAAAA

iRFP670

GGGCGAAUUAAGAGAGAAAAGAAGAGUAAGAAGAAAUAUAAGACACCGGUCGCCACCAUGGCGCGUAAGGUCGAUCUCACCUCCUGCGAUCGCGAGCCGAUCCACAUCCCCGGCAGCAUUCAGCCGUGCGGCUGCCUGCUAGCCUGCGACGCGCAGGCGGUGCGGAUCACGCGCAUUACGGAAAAUGCCGGCGCGUUCUUUGGACGCGAAACUCCGCGGGUCGGUGAGCUACUCGCCGAUUACUUCGGCGAGACCGAAGCCCAUGCGCUGCGCAACGCACUGGCGCAGUCCUCCGAUCCAAAGCGACCGGCGCUGAUCUUCGGUUGGCGCGACGGCCUGACCGGCCGCACCUUCGACAUCUCACUGCAUCGCCAUGACGGUACAUCGAUCAUCGAGUUCGAGCCUGCGGCGGCCGAACAGGCCGACAAUCCGCUGCGGCUGACGCGGCAGAUCAUCGCGCGCACCAAAGAACUGAAGUCGCUCGAAGAGAUGGCCGCACGGGUGCCGCGCUAUCUGCAGGCGAUGCUCGGCUAUCACCGCGUGAUGUUGUACCGCUUCGCGGACGACGGCUCCGGGAUGGUGAUCGGCGAGGCGAAGCGCAGCGACCUCGAGAGCUUUCUCGGUCAGCACUUUCCGGCGUCGCUGGUCCCGCAGCAGGCGCGGCUACUGUACUUGAAGAACGCGAUCCGCGUGGUCUCGGAUUCGCGCGGCAUCAGCAGCCGGAUCGUGCCCGAGCACGACGCCUCCGGCGCCGCGCUCGAUCUGUCGUUCGCGCACCUGCGCAGCAUCUCGCCCUGCCAUCUCGAAUUUCUGCGGAACAUGGGCGUCAGCGCCUCGAUGUCGCUGUCGAUCAUCAUUGACGGCACGCUAUGGGGAUUGAUCAUCUGUCAUCAUUACGAGCCGCGUGCCGUGCCGAUGGCGCAGCGCGUCGCGGCCGAAAUGUUCGCCGACUUCUUAUCGCUGCACUUCACCGCCGCCCACCACCAACGCAGAUCUCAUAUGCAUCUCGAGUGAUAGUCUAGACCUUCUGCGGGGCUUGCCUUCUGGCCAUGCCCUUCUUCUCUCCCUUGCACCUGUACCUCUUGGUCUUUGAAUAAAGCCUGAGUAGGAAAAAAAAAAAAAAAAAAAAAAAAAAAAAAAAAAAAAAAAAAAAAAAAAAAAAAAAAAAAAAAAAAAAAAAAAAAAAAAAAAAAAAAAAAAAAAAAAAAAAAAAAAAAAAAAAAAAAAAA

U1utr-EGFP （Protein binding motif）

GACAGCAUUGUACCCAGAGUCUGUCCCCAGACAUUGCACCUGGCGCUGUCCGCAGAUCGAGAAGAAGGCGAAUUAAGAGAGAAAAGAAGAGUAAGAAGAAAUAUAAGACACCGGUCGCCACCAUGGUGAGCAAGGGCGAGGAGCUGUUCACCGGGGUGGUGCCCAUCCUGGUCGAGCUGGACGGCGACGUAAACGGCCACAAGUUCAGCGUGUCCGGCGAGGGCGAGGGCGAUGCCACCUACGGCAAGCUGACCCUGAAGUUCAUCUGCACCACCGGCAAGCUGCCCGUGCCCUGGCCCACCCUCGUGACCACCCUGACCUACGGCGUGCAGUGCUUCAGCCGCUACCCCGACCACAUGAAGCAGCACGACUUCUUCAAGUCCGCCAUGCCCGAAGGCUACGUCCAGGAGCGCACCAUCUUCUUCAAGGACGACGGCAACUACAAGACCCGCGCCGAGGUGAAGUUCGAGGGCGACACCCUGGUGAACCGCAUCGAGCUGAAGGGCAUCGACUUCAAGGAGGACGGCAACAUCCUGGGGCACAAGCUGGAGUACAACUACAACAGCCACAACGUCUAUAUCAUGGCCGACAAGCAGAAGAACGGCAUCAAGGUGAACUUCAAGAUCCGCCACAACAUCGAGGACGGCAGCGUGCAGCUCGCCGACCACUACCAGCAGAACACCCCCAUCGGCGACGGCCCCGUGCUGCUGCCCGACAACCACUACCUGAGCACCCAGUCCGCCCUGAGCAAAGACCCCAACGAGAAGCGCGAUCACAUGGUCCUGCUGGAGUUCGUGACCGCCGCCGGGAUCACUCUCGGCAUGGACGAGCUGUACAAGUAGUCUAGACCUUCUGCGGGGCUUGCCUUCUGGCCAUGCCCUUCUUCUCUCCCUUGCACCUGUACCUCUUGGUCUUUGAAUAAAGCCUGAGUAGGAAAAAAAAAAAAAAAAAAAAAAAAAAAAAAAAAAAAAAAAAAAAAAAAAAAAAAAAAAAAAAAAAAAAAAAAAAAAAAAAAAAAAAAAAAAAAAAAAAAAAAAAAAAAAAAAAAAAAAAA

MS2CP(WT)

GGGCGAAUUAAGAGAGAAAAGAAGAGUAAGAAGAAAUAUAAGACACCGGUCGCCACCAUGGCUUCUAACUUUACUCAGUUCGUUCUCGUCGACAAUGGCGGAACUGGCGACGUGACUGUCGCCCCAAGCAACUUCGCUAACGGGGUCGCUGAAUGGAUCAGCUCUAACUCGCGAUCACAGGCUUACAAAGUAACCUGUAGCGUUCGUCAGAGCUCUGCGCAGAAUCGCAAAUACACCAUCAAAGUCGAGGUGCCUAAAGGCGCAUGGAGGUCUUACUUAAAUAUGGAACUAACCAUUCCAAUUUUCGCCACGAAUUCCGACUGCGAGCUUAUUGUUAAGGCAAUGCAAGGUCUCCUAAAAGAUGGAAACCCGAUUCCCUCGGCCAUCGCGGCCAACUCCGGCAUCUACUGAAUCUAGACCUUCUGCGGGGCUUGCCUUCUGGCCAUGCCCUUCUUCUCUCCCUUGCACCUGUACCUCUUGGUCUUUGAAUAAAGCCUGAGUAGGAAAAAAAAAAAAAAAAAAAAAAAAAAAAAAAAAAAAAAAAAAAAAAAAAAAAAAAAAAAAAAAAAAAAAAAAAAAAAAAAAAAAAAAAAAAAAAAAAAAAAAAAAAAAAAAAAAAAAAAA

U1A(full)

GGGCGAAUUAAGAGAGAAAAGAAGAGUAAGAAGAAAUAUAAGACACCGGUCGCCACCAUGGcgGCAGUUCCCGAGACCCGCCCUAACCACACUAUUUAUAUCAACAACCUCAAUGAGAAGAUCAAGAAGGAUGAGCUAAAAAAGUCCCUGUACGCCAUCUUCUCCCAGUUUGGCCAGAUCCUGGAUAUCCUGGUAUCACGGAGCCUGAAGAUGAGGGGCCAGGCCUUUGUCAUCUUCAAGGAGGUCAGCAGCGCCACCAACGCCCUGCGCUCCAUGCAGGGUUUCCCUUUCUAUGACAAACCUAUGCGUAUCCAGUAUGCCAAGACCGACUCAGAUAUCAUUGCCAAGAUGAAAGGCACCUUCGUGGAGCGGGACCGCAAGCGGGAGAAGAGGAAGCCCAAGAGCCAGGAGACCCCGGCCACCAAGAAGGCUGUGCAAGGCGGGGGAGCCACCCCCGUGGUGGGGGCUGUCCAGGGGCCUGUCCCGGGCAUGCCGCCGAUGACUCAGGCGCCCCGCAUUAUGCACCACAUGCCGGGCCAGCCGCCCUACAUGCCGCCCCCUGGUAUGAUCCCCCCGCCAGGCCUUGCACCUGGCCAGAUCCCACCAGGGGCCAUGCCCCCGCAGCAGCUUAUGCCAGGACAGAUGCCCCCUGCCCAGCCUCUUUCUGAGAAUCCACCGAAUCACAUCUUGUUCCUCACCAACCUGCCAGAGGAGACCAACGAGCUCAUGCUGUCCAUGCUUUUCAAUCAGUUCCCUGGCUUCAAGGAGGUCCGUCUGGUACCCGGGCGGCAUGACAUCGCCUUCGUGGAGUUUGACAAUGAGGUACAGGCAGGGGCAGCUCGCGAUGCCCUGCAGGGCUUUAAGAUCACGCAGAACAACGCCAUGAAGAUCUCCUUUGCCAAGAAGUAGUCUAGACCUUCUGCGGGGCUUGCCUUCUGGCCAUGCCCUUCUUCUCUCCCUUGCACCUGUACCUCUUGGUCUUUGAAUAAAGCCUGAGUAGGAAAAAAAAAAAAAAAAAAAAAAAAAAAAAAAAAAAAAAAAAAAAAAAAAAAAAAAAAAAAAAAAAAAAAAAAAAAAAAAAAAAAAAAAAAAAAAAAAAAAAAAAAAAAAAAAAAAAAAAA

**Supplementary References**

1. Ono, H., Kawasaki, S. and Saito, H., (2019) Orthogonal Protein-Responsive mRNA Switches for Mammalian Synthetic Biology. *ACS Synth Biol.*. **9**, 169-174

2. Jovine, L., Oubridge, C., Avis, J.M. and Nagai, K. (1996) Two structurally different RNA molecules are bound by the spliceosomal protein U1A using the same recognition strategy. *Structure*. **4**, 621-31.

3. Kawasaki, S., Fujita, Y., Nagaike, T., Tomita, K. and Saito, H. (2017) Synthetic mRNA devices that detect endogenous proteins and distinguish mammalian cells. *Nucleic Acids Res*. **45**, e117.
